# Comparison of imaging-based bone marrow dosimetry methodologies and their dose–effect relationships in [^177^Lu]Lu-PSMA-617 RLT including a novel method with active marrow localization

**DOI:** 10.1186/s40658-025-00816-6

**Published:** 2025-12-04

**Authors:** Avery B. Peterson, Scott J. Wilderman, Johan Blakkisrud, Ka Kit Wong, Kirk A. Frey, Yuni K. Dewaraja

**Affiliations:** 1 Department of Radiology, University of Michigan, Ann Arbor, MI 48109 USA; 2 Department of Radiation Oncology, Wayne State University, Detroit, MI USA; 3 Department of Nuclear Engineering and Radiological Sciences, University of Michigan, Ann Arbor, MI USA; 4 Department of Physics and Computational Radiology, Oslo University Hospital, Oslo, Norway

**Keywords:** 177Lu, PSMA, Radioligand therapy, Bone marrow, Dosimetry

## Abstract

**Purpose:**

Establishing accurate methods for red marrow (RM) dosimetry is an important step toward patient-specific treatment guidance. We compared image-based dosimetry methods and investigated their role in predicting changes in blood counts following [^177^Lu]Lu-PSMA-617 radioligand therapy (^177^Lu RLT).

**Methods:**

Four image-based dosimetry methodologies were applied to patients who received 2-bed position serial ^177^Lu SPECT/CT after cycle 1 of RLT, with segmentation of all spongiosa within the field-of-view performed on CT using deep learning tools. Cycle 1 RM absorbed doses (ADs) were estimated with: 1) the time-integrated activity (TIA) in segmented spongiosa coupled with MIRD-based S-values (MIRD); 2) the TIA concentration in the segmented aorta (a surrogate for blood-based dosimetry) coupled with MIRD-based S values (MIRD_aorta_); 3) the voxel-level TIA map coupled with an in-house Monte Carlo (MC) dosimetry code that incorporated a micro-scale modeling of the spongiosa (MC); and 4) a novel method that utilizes [^68^Ga]Ga-PSMA-11 PET/CT and [^99m^Tc]Tc-sulfur colloid (SC) SPECT/CT for tumor and marrow localization coupled with the above MC code, modified to allow tumor infiltration of the spongiosa (MC_SC+PET_). Spearman rank correlation of AD from the four methods with changes in select blood counts was evaluated.

**Results:**

Imaging data was available for 20 patients for methods 1–3, while SC images were available for 12 patients for method 4. Cycle 1 AD to the FOV RM was, on average, 1.9 Gy (range: 0.1–8.0 Gy) for MIRD, 0.08 Gy (range: 0.01–0.27 Gy) for MIRD_aorta_, 2.5 Gy (range: 0.1–10.3 Gy) for MC, and 1.6 Gy (range: 0.1–4.6 Gy) for MC_SC+PET_. The ADs from MIRD_aorta_ were not concordant with MIRD, MC, or MC_SC+PET_ (|CCC|< 0.01) and were generally underestimates. For 3 patients with high bone tumor burden, MC_SC+PET_ gave lower average AD than MIRD (39%) and MC (53%), potentially due to more accurate localization of marrow and tumor. Cycle 1 RM ADs were correlated with relative change in blood counts at 6-weeks post-cycle 1 with significant correlation observed for neutrophils with MIRD, MC, and MC_SC+PET_ with Spearman rank correlations ranging from *r* = − 0.61 to *r* = − 0.88 (*P* < 0.01). Correlation with white blood cells at 6-months was also significant with *r* = − 0.80 (*P* < 0.01) for these three methods. MIRD_aorta_ did not correlate with any acute or chronic changes in blood counts.

**Conclusion:**

The RM AD estimates from the blood-based surrogate were not concordant with the other image-based calculations and did not correlate with changes in blood values. Including patient-specific tumor and marrow distribution information resulted in lower AD for patients with a high bone metastatic burden. These findings have implications for managing hematological toxicities in ^177^Lu RLT, especially if dosimetry-guided treatment planning is considered.

**Supplementary Information:**

The online version contains supplementary material available at 10.1186/s40658-025-00816-6.

## Introduction

[^177^Lu]Lu-PSMA-617 is not expected to be taken up in hematopoietically active marrow cells or blood [1, 2], thus there have been studies that calculate absorbed dose (AD) to bone marrow (BM) using blood-based estimates [3–6]. However, metastatic castrate resistant prostate cancer (mCRPC) patients often present with bone metastases, affecting > 90% of patients over the course of the disease [7]. Thus, imaging-based methods for [^177^Lu]Lu-PSMA-617 therapy have also been employed [8, 9] and may be necessary to account for the absorbed dose from PSMA-avid disease impinging on areas of active BM. Furthermore, encroaching disease can displace hematopoietically active cells [8, 10, 11] and create heterogeneous distributions of red marrow (RM) that differ from the standard models that are used in traditional S-value calculations.

The incidence of Common Terminology Criteria for Adverse Events (CTCAE) grade 3 + hematological toxicity was 11% thrombocytopenia, 8% anemia in the phase 2 TheraP trial [12] and 8% thrombocytopenia, 13% anemia in the phase 3 VISON trial [13]. An additional retrospective study of 40 patients indicated a higher incidence of 25% thrombocytopenia and 22% anemia [14]. While not common, the presence of hematological toxicity motivates the need to establish dose-toxicity relationships and accurate quantification of AD to active marrow, and is likely dependent on knowledge of the patient-specific distribution of active marrow and tumor.

Motivated by the limited data available on BM dosimetry following [^177^Lu]Lu-PSMA radioligand therapy (RLT) and its relevance for safety, in this study, we compare patient results derived from four [^177^Lu]Lu-PSMA-617 SPECT/CT image-based BM dosimetry methods. This includes a relatively simple, approach, yet to be investigated in RLT, that uses SPECT-derived counts in the aorta as a surrogate for ex-vivo blood sampling, and a more sophisticated novel approach integrating tumor and marrow distribution information from [^68^Ga]Ga-PSMA-11 PET/CT and [^99m^Tc]Tc-sulfur colloid SPECT/CT with a macro/micro model of the spongiosa.

## Methods

### Patients

Twenty patients who underwent at least one cycle of ^177^Lu RLT for treatment of mCRPC at the University of Michigan Hospital and received serial post-therapy SPECT/CT imaging following the first cycle were included in this study. Patients provided written informed consent in accordance with the local Institutional Review Board-approved study protocol or were included retrospectively, if dosimetric imaging was performed for clinical indications. Patients were injected with an average of 7.07 GBq (range: 5.84–7.52 GBq) [^177^Lu]Lu-PSMA-617 in cycle 1. Relevant blood toxicity markers were collected prior to the start of therapy and before the start of each additional cycle of therapy. The time between cycle 1 and cycle 2 was nominally 6 weeks and ranged from 41–43 days for 19 patients (one patient received cycle 2 at 84 days). Baseline blood counts and additional patient characteristics are summarized in Supplemental Table 1 (Online Resource 1).

### Quantitative ^177^Lu SPECT/CT and time-integrated activity map generation

Following the first cycle of therapy, 2-bed position (nose through mid-thigh) [^177^Lu]Lu-PSMA-617 SPECT/CT imaging was performed at 3 (5/20) or 4 (15/20) timepoints at approximately 1 h, 1–2 d, 3–4 d, and/or 5–6 d on an Intevo Bold system (Siemens Healthineers, Munich, Germany). Each bed position was acquired for 18 min (2 heads, 60 views/head, 18 s/view) with a medium energy collimator. The acquisition window was 20% at the 208 keV photopeak with 10% scatter windows. Quantitative reconstruction using xSPECT software provided images in units of Bq/mL. The CT of the SPECT/CT was acquired at 110 kVp and 60 mAs for the 1–2 d acquisition (considered the “reference” time point) while all other time points were acquired at a lower exposure. Further imaging details are given in Supplemental Table 2 (Online Resource 1). The multi-timepoint ^177^Lu SPECT images were coregistered to the reference time point image with a contour-intensity-based multiple local-rigid registration algorithm (MIM Software, Cleveland, OH, USA).

The coregistered SPECT images were used to fit monoexponential or 3-parameter biexponential time-activity curves (TACs) on a voxel-level. The fit with the lower Akaike information criterion was considered the best fit and used for that voxel [15]. Time-integrated activity (TIA) maps were computed through analytic integration. TIA maps were resampled to the CT grid size for use with our in-house Monte Carlo (MC) dosimetry code [16].

### Spongiosa segmentation

**Fig. 1:**
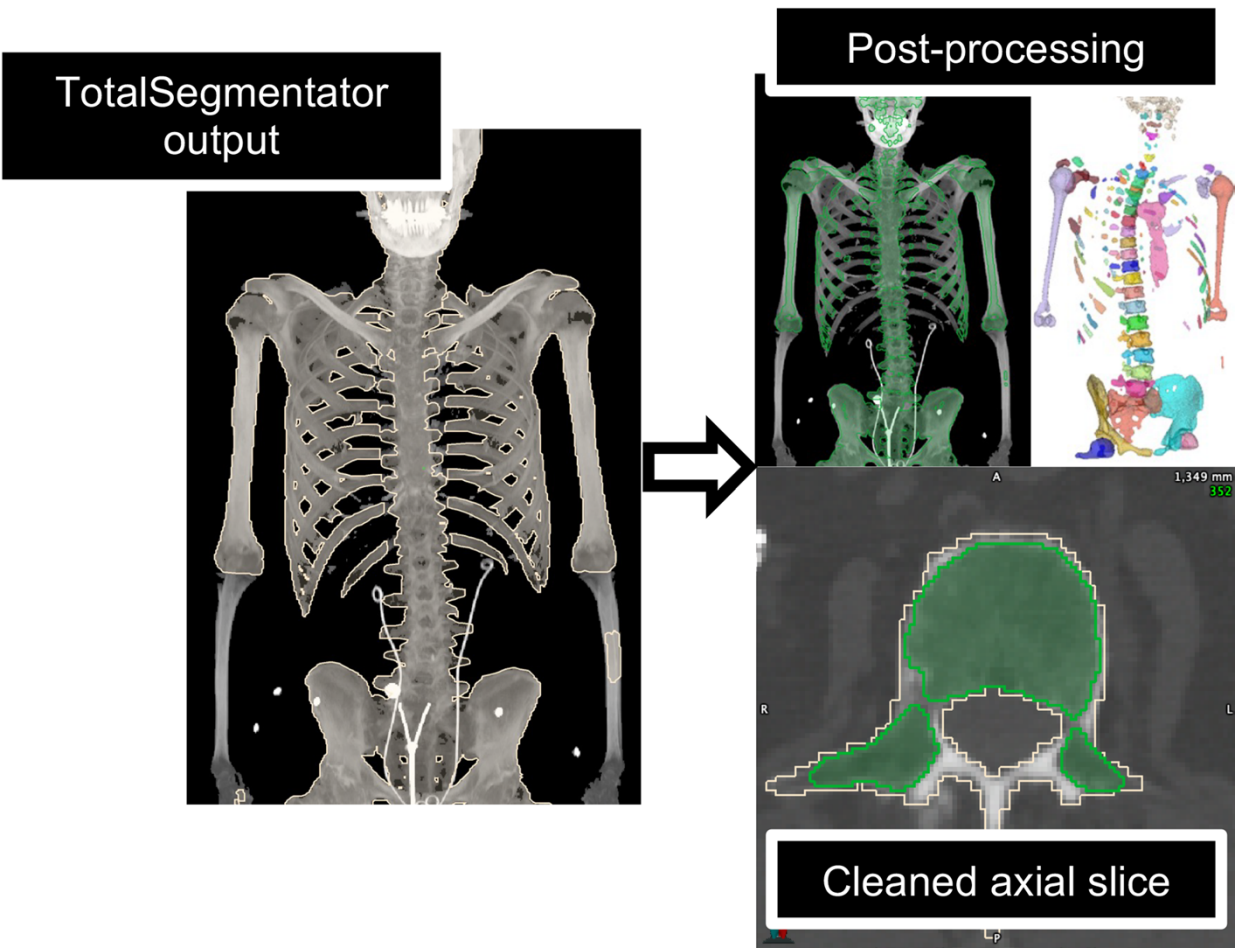
The output of automatic segmentation via TotalSegmentator (beige) and the resulting spongiosa-only contour after automatic and manual refinement (green). A 3D visualization of the spongiosa segmentation is also displayed

Marrow-bearing skeletal sites were segmented on the reference CT (of ^177^Lu SPECT/CT) for each patient semi-automatically. Segmentation began with deep learning-based automatic segmentation of normal organs and skeletal sites with the open-source model, TotalSegmentator [17, 18] (version 2.0.5), at full resolution (1.5 mm × 1.5 mm × 1.5 mm). These masks were further refined using an in-house automatic workflow consisting of contraction, subthresholding, and morphological operations to exclude cortical bone (Fig. 1, Supplemental Table 3 ). Skeletal sites were adjusted manually if necessary and visually approved.

### Image-based MIRD (MIRD) dosimetry

The segmented spongiosa, potentially including pathologic infiltration, and TIA maps were used to perform image-based BM dosimetry calculations using self-dose S-values and the MIRD schema [19]. This methodology will be referred to as “MIRD.” The total TIA summed across all delineated skeletal spongiosa regions was multiplied by a skeletal average S-value with a mass scaling factor:

1 $$ D_{MIRD} = \tilde{A} \times S_{RM \leftarrow RM} \times \frac{{m_{{RM,{\text{phantom}}}} }}{{m_{RM} }} $$

With $\widetilde{A}$ the total spongiosa TIA in units of MBq-sec, ${S}_{RM\leftarrow RM}=1.07\times {10}^{-5} \frac{mGy}{MBq-sec}$ is the RM to RM S-value [20], ${m}_{RM,phantom}={\rho }_{RM}\times {v}_{RM,phantom}$ the mass corresponding to the University of Florida reference man phantom (${v}_{RM,phantom}=1170 mL$) with known RM density ${\rho }_{RM}=1.03 g/mL$ [21], and ${m}_{RM}={{\rho }_{RM}\times v}_{RM}\times {f}_{RM}$ the patient’s RM mass in the FOV estimated from the segmented spongiosa volume, ${v}_{RM}$, and the skeletal average RM volume fraction, ${f}_{RM}=0.52$, provided by Hemmingsson et al. [20].

### Imaging-derived blood-based MIRD (MIRD_aorta_) dosimetry

Most previous efforts to quantify AD to the RM in RLT have utilized methods based on blood sampling [3–6] because it is assumed that there is no specific uptake of [^177^Lu]Lu-PSMA-617 in marrow cells [1, 2]. No blood samples were available in our study to measure the activity concentration in blood. However, as a surrogate, we measured the mean activity concentration in VOIs (from TotalSegmentator) defined on the aorta of each ^177^Lu SPECT scan, ${[A]}_{aorta}$. A similar methodology has been employed previously by Hemmingsson et al. [20] and was adapted from methods used in ^18^F PET imaging [22], which indicates blood sampling is correlated with imaging counts to an average accuracy of 4.8% ± 8.6%. Given the bi-phasic nature of the blood time-activity curves [23], a four-parameter bi-exponential was used to fit the serial data. This fit function was integrated to determine blood time-integrated activity concentration, ${\left[\widetilde{A}\right]}_{aorta}$.

The RM to blood activity concentration ratio (RMBLR) was used to account for the difference in plasma concentration between the blood in the aorta and the fluid in the marrow extracellular space [24] under the assumption that [^177^Lu]Lu-PSMA-617 does not have specific uptake in RM. The RMBLR is computed for each individual patient based on their baseline hematocrit (HCT). Thus, the time-integrated activity concentration per mass of RM ${\left[\widetilde{A}\right]}_{RM}$ was computed as:

2 $$ \left[ {\tilde{A}} \right]_{RM} = \left[ {\tilde{A}} \right]_{{{\text{aorta}}}} \times RMBLR = \left[ {\tilde{A}} \right]_{{{\text{aorta}}}} \times \frac{0.19}{{1 - HCT}} $$

The resulting aorta-blood-based AD estimate can be calculated with:

3 $$ D_{{MIRD_{{{\text{aorta}}}} }} = \left[ {\tilde{A}} \right]_{RM} \times m_{RM} \times S_{RM \leftarrow RM} $$

where ${S}_{RM\leftarrow RM}$ is the self-dose S-value used in the previous MIRD calculation (Eq. 1).

### Computing bone volume fraction for MC methods

For comparison with MIRD-based AD calculations, we leveraged our in-house MC dosimetry code to account for the effect of varying voxel material composition on a micro-scale. The bone volume fraction (BVF) in the spongiosa was defined as the ratio of the total volume of trabecular bone to the total volume of spongiosa. For our MC dosimetry, we utilized a three-compartment spongiosa model that considers bone, yellow marrow (YM), and RM. Within this framework:

4 $$ BVF = \frac{{v_{{{\text{bone}}}} }}{{v_{RM} + v_{YM} + v_{{{\text{bone}}}} }} $$

where ${v}_{bone}$, ${v}_{RM}$, and ${v}_{YM}$ are the volumes of hard bone, RM, and YM, respectively. Given the cellularity fraction (CF), which is the volume of marrow that is made of RM ($CF=\frac{{v}_{RM}}{{v}_{RM}+{v}_{YM}})$, within our three-compartment model we compute the BVF for any voxel given its density and CF, such that:

5 $$ \rho_{{{\text{marrow}},i}} = CF_{i} \times \rho_{RM} + \left( {1 - CF_{i} } \right) \times \rho_{YM} $$

6 $$ BVF_{i} = \frac{{\rho_{{{\text{voxel}},i}} - \rho_{{{\text{marrow}},i}} }}{{\rho_{{{\text{bone}}}} - \rho_{{{\text{marrow}},i}} }} $$

With ${\rho }_{RM}=1.03 g/mL$, ${\rho }_{YM}=0.98 g/mL$, and ${\rho }_{bone}=1.92 g/mL$ the known densities of RM, YM, and trabecular bone, respectively [21]. The ${\rho }_{voxel,i}$ is the density of voxel $i$ and $C{F}_{i}$ is the CF of voxel $i$. CF was determined differently for each MC calculation methodology below. The ${\rho }_{voxel,i}$ was obtained from the reference CT with a bi-linear function derived from calibration measurement that maps HU to physical density [25].

### Image-based Monte Carlo (MC) dosimetry with micro-scale spongiosa model

MC voxel dosimetry was performed with a version of the Dose Planning Method (DPM) MC code [26] that has been previously adapted for internally-administered radionuclides [16] and then further extended to include a novel method for dosimetry in marrow-bearing regions of bone [27]. DPM has been extensively benchmarked against both other MC programs as well as experimental measurements [28].

Direct RM dosimetry in DPM is accomplished by employing pre-computed look-up tables of electron energy absorption fractions (EAFs) to apportion electron dose to constituent bone voxel components (RM, YM, and trabecular bone) as a function of electron energy, bone volume fraction, RM cellularity, and source of electron (i.e., initial beta, boundary crossing, or secondary particle) whenever an electron is introduced into a spongiosa region [27] (Look-up tables in Online Resource 2). In this three-component model of the spongiosa, we assumed activity originated from the active RM. Outside the spongiosa, the transport mechanics and material composition remain unchanged from that in the macro DPM algorithm [16, 26] with voxels assumed to have a homogeneous material composition (air or water) with density from the CT-derived density map. MC simulations with DPM used 10^8^ histories and ran for less than 10 min on an 8-core processor operating at 2.4 GHz. This dosimetry method will henceforth be denoted as “MC.”

**Fig. 2:**
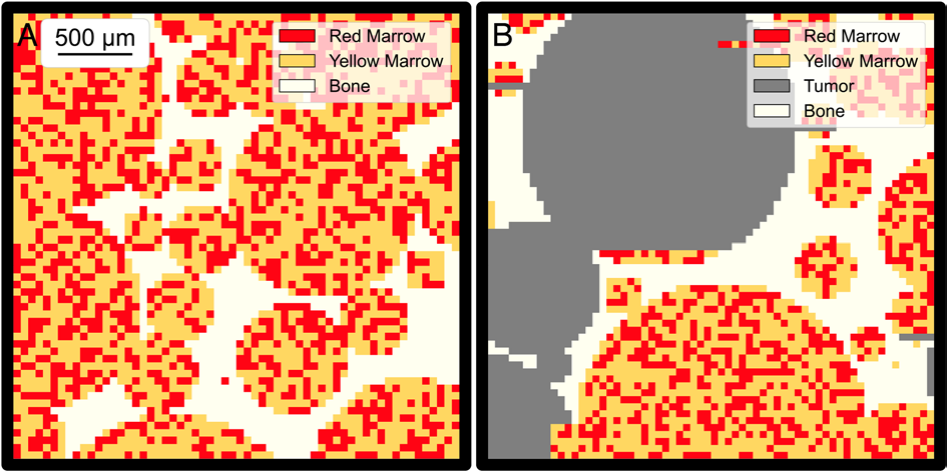
Examples of micro-scale spongiosa model used for tabular electron EAF data generation in EGS5 for A) our three-compartment model and B) our four-compartment model used in MC_SC+PET_. Both images are for 17% BVF and 40% CF

In this work we extend the prior energy apportioning methodology to account for the presence of lesions inside spongiosa regions. First, additional energy absorption fraction tables were generated for four-component (bone, RM, YM, and lesion) spongiosa (Online Resource 2). As in [27], we again used the general-purpose EGS5 code [29], modifying the original three-component simulated trabecular bone model to include lesion regions. Representative geometries for the three-component and four-component models are shown in Fig. 2A and B, for 50 µm voxels. RM and YM subvoxels in the micro-scale geometry are assigned randomly in proportions dictated by the CF.

EAFs are compiled for 12 BVFs (3–35%), 11 CFs (1–99%), and 37 energies (10 keV–2.5 MeV) to ensure minimal interpolation error. Pre-defined spongiosa regions including the clavicles, femurs, hips, humeri, ribs, sacrum, scapulae, skull, sternum, and thoracic, lumbar, and cervical spine were delineated using the semi-automatic procedure described above. For this approach, we do not attempt to consider patient-specific bone marrow or tumor distributions, as such, reference values for CF must be used and regions of spongiosa were identified and assigned a CF based on Table 41 in ICRP 70 [30]. The BVF of each voxel in a marrow-bearing region was computed based on the CF assigned to each skeletal region mask and the CT-derived density according to Eq. 6.

### Image-based MC dosimetry with sulfur-colloid imaging (MC_SC+PET_) to inform micro-scale spongiosa model

#### Active marrow quantification and tumor localization

In a novel methodology that will be referred to as “MC_SC+PET_,” we utilized pre-therapy [^68^Ga]Ga-PSMA-11 PET/CT and [^99m^Tc]Tc-sulfur colloid SPECT/CT to assist with patient-specific localization and quantification of tumor and marrow, respectively. This approach was possible for 12/20 patients, due to the limited availability of sulfur colloid images.

Pre-therapy, whole-body [^68^Ga]Ga-PSMA-11 PET/CTs performed according to the standard of care for ^177^Lu RLT were available in units of SUV_bw_. Imaging details are in Supplemental Table 2 as the [^68^Ga]Ga-PSMA-11 PET/CT scans were sometimes performed with different scan parameters or at other institutions due to the diagnostic nature of the acquisition. Twelve patients underwent [^99m^Tc]Tc-sulfur colloid SPECT/CT imaging either the week before the first cycle of treatment (*N* = 2) or after the first cycle (*N* = 10) with a median time since start of treatment of 39 days (range: 28–92 days). Each patient was administered a median dose of 378 MBq (range: 339–421 MBq) [^99m^Tc]Tc-sulfur colloid (Sun Pharmaceutical Industries, Billerica, MA, USA). To match the ^177^Lu SPECT/CT FOV, ^99m^Tc SPECT/CT scans were also 2 bed positions, with each position acquired for 12 min (2 heads, 60 views/head, 12 s/view) and performed on the Intevo Bold and reconstructed quantitatively. Previous studies in phantoms indicate that, on average, absolute quantification accuracy within 5% can be achieved for ^99m^Tc SPECT imaging for spheres down to 0.5 mL with partial volume correction [31]. [^99m^Tc]Tc-sulfur colloid SPECT/CTs for 3 patients representative of low, medium, and high marrow reserves are provided in Fig. S1 (Online Resource 1).

The ^68^Ga PET/CT and ^99m^Tc SPECT/CT scans were deformably coregistered with a CT-CT deformation utilizing Elastix via itkElastix, with deformation parameters provided in Online Resource 3. Deformations were validated visually for reasonableness, particularly with respect to marrow-bearing regions. The functional image of each study had the same deformation applied followed by multiplication with the determinant of the spatial Jacobian matrix at each voxel to preserve activity in the image [32]. These deformations mapped ^68^Ga PET/CT and ^99m^Tc SPECT/CT to the reference ^177^Lu CT image and all images were resampled to the matrix and voxel size of the CT of the ^177^Lu SPECT/CT.

The pre-therapy ^68^Ga PET scans were used to identify regions of pathologic uptake within the skeleton, assumed to be tumor, using a SUV threshold of 3 [33]. The tumor segmentations were transferred from ^68^Ga PET/CT to the CT of the reference ^177^Lu SPECT/CT after the ^68^Ga PET/CT series had been deformably coregistered to the ^177^Lu SPECT/CT series.

^99m^Tc-SC SPECT imaging was used to quantify the mass of RM in each voxel of delineated skeletal regions. First, a region that showed strong, uniform uptake on ^99m^Tc-SC SPECT imaging but relatively weak and non-focal uptake on [^68^Ga]Ga-PSMA PET imaging was identified qualitatively and selected as the reference marrow region for the given patient. The median ^99m^Tc-SC activity concentration of this reference region was 7.8 kBq/mL (range: 3.3–42.9 kBq/mL). This region was assumed to have a physiologically normal marrow space with no tumor infiltration and thus a CF for normal subjects taken from the literature was assigned to the reference region. We utilized the RM and YM volume fractions of Dunnill et al. [34] of healthy L2 vertebrae for the computation of CF as a function of age (Fig. S2 in Online Resource 1). Given the reference CF, the CT-derived density map, and the reference region mask, the mass of RM in the reference region was given by:

7 $$ m_{RM,ref} = \left( {1 - BVF_{ref} } \right) \times CF_{ref} \times v_{ref} \times \rho_{RM} $$

where $BV{F}_{ref}$ is the average BVF in the reference region (using Eq. 6), ${m}_{RM,ref}$ is the RM mass in the reference region, $C{F}_{ref}$ is the age-based CF calculated for the patient, ${v}_{ref}$ is the total volume of the reference region, and ${\rho }_{RM}=1.03 g/mL$ the known density of RM.

The RM mass was divided by the activity concentration in the reference region to determine a ^99m^Tc-SC activity concentration per RM mass scaling factor (SF) which could be applied to the ^99m^Tc-SC activity concentration map in every skeletal region to obtain an RM mass distribution that indicates the mass of RM present in each voxel of spongiosa ${m}_{RM,i}$:

8 $$ SF = \frac{{m_{RM,ref} }}{{\left[ {A_{SC,ref} } \right]}} $$

9 $$ m_{RM,i} = \left[ {A_{SC,i} } \right] \times SF $$

#### Micro-scale Monte Carlo

**Fig. 3:**
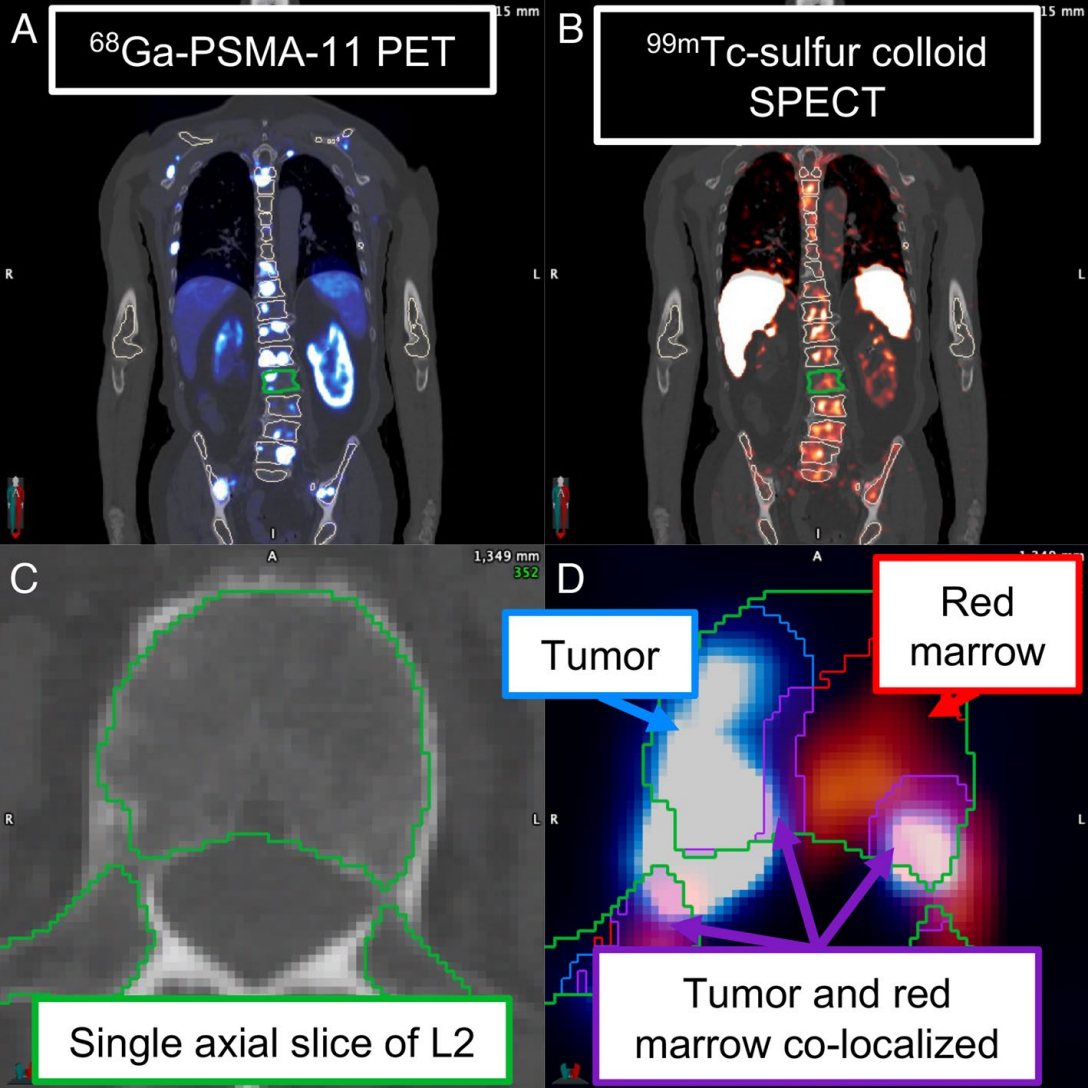
**A** [^68^Ga]Ga-PSMA-11 PET indicating disease distribution and **B** [^99m^Tc]Tc-sulfur colloid SPECT indicating marrow distribution for patient P2 with **C** the anatomy and segmentation (green) of a single axial slice of L2 and **D** the complementary nature of the two scans with voxels containing tumor (blue), RM (red), or both (purple) indicated

Scoring routines in the RM version of DPM [27] were also adapted to accommodate four-component spongiosa regions. We further modified our DPM implementation to use ^99m^Tc-SC SPECT images for RM quantification and to allow for tumor localization via [^68^Ga]Ga-PSMA PET. BVF and the CF of a voxel were determined given the CT-derived density and RM mass of the voxel (Eq. 9) as detailed in Online Resource 4. In the absence of tumor (localized using ^68^Ga PET), the same electron EAFs as a function of BVF and CF are used as with our first MC method. If the tumor mask generated by thresholding the ^68^Ga PET scan overlapped voxels that contain RM according to the ^99m^Tc-SC scan (Fig. 3), both RM and tumor were assumed to occupy those voxels. Due to the difficulty and uncertainty in estimating the fraction of encroaching tumor within a voxel, we assumed an equal volume of tumor and marrow (RM + YM). New electron EAFs for a four-compartment model (tumor, RM, YM, and bone) were generated in much the same way as described previously, with a micro-scale model of the spongiosa structure, but with 50% of the non-bone spongiosa volume occupied by tumor subvoxels (Fig. 2B). Tumor was assigned a mass density of 1.00 g/mL with tissue composition from Maughan et al. [35] and all activity was assumed to originate from specific uptake in the tumor subvoxels. Online Resource 4 contains details of the BVF and CF calculations in the presence of tumor. DPM MC simulations for the MC_SC+PET_ methodology used 10^8^ histories and ran for less than 10 min on an 8-core processor operating at 2.4 GHz. Note that a flowchart summarizing the inputs to DPM for both the MC and MC_SC+PET_ methodologies is given in Fig. S3 in Online Resource 1.

#### TIA correction to account for partial volume effects

Counts spill-in from tumor voxels to marrow voxels due to limited SPECT resolution can lead to substantial overestimation of marrow activity. The bone metastases responsible for this effect are often small and can be located at the periphery of a given skeletal site, leading to difficulty applying volume-based recovery-coefficients to add counts to tumor and remove spill-in from marrow voxels. So, to mitigate these partial volume effects, we leveraged the TIA concentration in RM, ${\left[\widetilde{A}\right]}_{RM}$, computed for each patient from blood-pool imaging of the aorta as described previously, to preprocess the ^177^Lu TIA map before input to the MC dosimetry code with an in-house python script. This method implicitly assumes that the aorta contour is less affected by partial volume effects due to its relatively large, contiguous volume. First, the blood-based expected TIA for each marrow voxel was determined by multiplying ${\left[\widetilde{A}\right]}_{RM}$ (Eq. 2) by the marrow mass for that voxel as determined from the [^99m^Tc]Tc-sulfur colloid SPECT image (Eq. 9). Then, a comparison is made between this aorta-blood estimated TIA and the TIA estimated from serial ^177^Lu SPECT/CT imaging across all marrow-bearing voxels of each delineated skeletal region. If the aorta-blood derived TIA is lower, then the difference is assumed to be spill-out and deposited into the tumor voxels of that region; if higher, then the aorta-blood derived TIA is used because the count statistics on the large aorta region is assumed to be better than the statistics of the blood counts in any single marrow-bearing region. These “corrected” maps are used for the MC_SC+PET_ AD calculation.

### Statistics

Dosimetry results across the four methods MIRD, MIRD_aorta_, MC, and MC_SC+PET_, were correlated with the relative percent change from baseline to approximately 6 weeks post-cycle 1 and 6 months after the start of therapy in blood-based toxicity markers. Correlations were evaluated using Spearman rank correlation coefficients. The different dosimetry methods were compared against each other using the concordance correlation coefficient (CCC). Statistical analysis was performed in Python 3.9.18 using Statsmodels 0.12.2 and Scipy 1.9.3. We assumed a significance level of P ≤ 0.05, but since each method was evaluated against 5 different toxicity markers, we applied the Bonferroni correction to account for multiple testing resulting in an adjusted significance level of *P* ≤ 0.01.

## Results

### Dosimetry

**Fig. 4:**
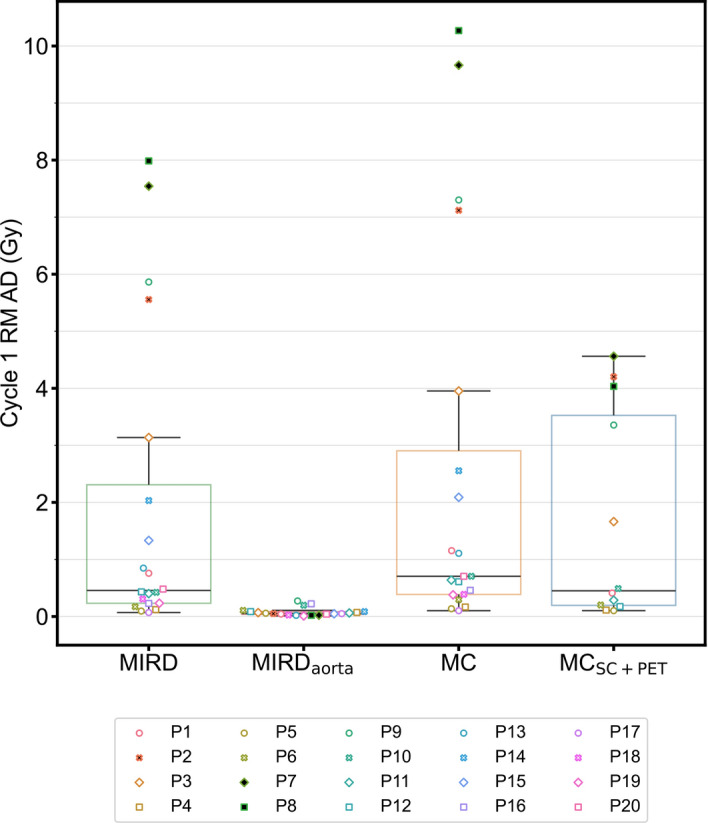
Box plots showing the AD to the entire FOV RM for 20 patients across 3 methodologies and 11 patients for the novel method requiring [^99m^Tc]Tc-SC imaging (MC_SC+PET_). Median values are represented by horizontal black lines. Symbols that are filled in black indicate patients (P2, P7, and P8) that had a high bone metastatic burden

AD to each patient’s entire RM from cycle 1 of therapy was on average 1.9 Gy for MIRD, 0.08 Gy for MIRD_aorta_, 2.0 Gy for MC, and 1.6 Gy for MC_SC+PET_. Complete summary statistics for each dosimetry method are presented in Table 1. Figure 4 shows each patient’s FOV RM AD across all four dosimetry methods as box plots and individual points. MIRD_aorta_ was not concordant with the other dosimetry methods (|CCC|< 0.01) and consistently resulted in lower ADs. MIRD and MC were moderately concordant with each other (CCC = 0.95), but not concordant with MC_SC+PET_ (CCC = 0.78 and CCC = 0.62, respectively). 3 patients (P2, P7, and P8) were identified with a high metastatic bone burden (> 10% of segmented spongiosa contains tumor) and among these 3 patients, the mean AD of MC_SC+PET_ is 39% lower than MIRD and 53% lower than MC (Fig. 4). Fig. S4 (Online Resource 1) additionally indicates the AD to each patient across the four methods, showing that MC_SC+PET_ ADs are much lower than MIRD and MC in some cases, particularly when heavy tumor infiltration of the skeleton is present. Among the other nine patients who had MC_SC+PET_ dosimetry, AD was on average 21% lower than MIRD and 47% lower than MC. For three patients, P5, P6, and P10, MIRD AD was lower than MC_SC+PET_ AD, with differences of 8%, 18%, and 16%, respectively. Individual time-activity curves corresponding to each patient’s aorta blood activity concentration used for MIRD_aorta_ dosimetry are presented in Fig. S5 (Online Resource 1). Additionally, AD to RM in individual skeletal regions is presented in Fig. S6 (Online Resource 1) for each patient and both the MC and MC_SC+PET_ dosimetry methods.

**Table 1:** Cycle 1 AD (Gy and Gy/GBq) statistics for all 4 dosimetry methods

Dosimetry Method	Mean	Std. Dev	Median	Minimum	Maximum
MIRD	1.90 (0.28)	2.63 (0.40)	0.46 (0.07)	0.07 (0.01)	7.99 (1.37)
MIRD_aorta_	0.08 (0.01)	0.07 (0.01)	0.05 (0.01)	0.01 (0.00)	0.27 (0.04)
MC	2.49 (0.36)	3.33 (0.50)	0.71 (0.11)	0.10 (0.01)	10.27 (1.76)
MC_SC+PET_	1.63 (0.24)	1.84 (0.27)	0.45 (0.06)	0.10 (0.01)	4.56 (0.69)

### Blood-based biomarkers

**Fig. 5:**
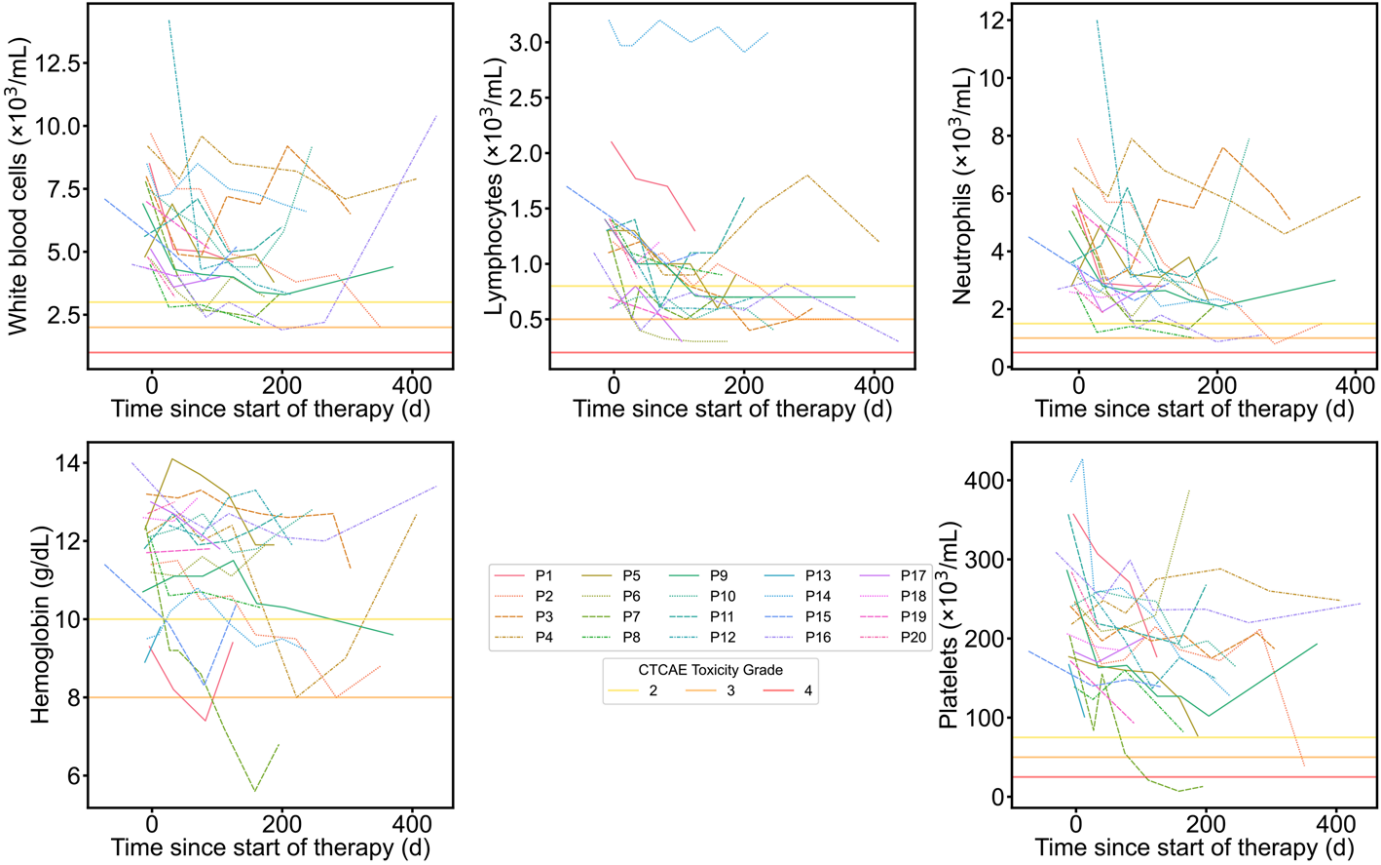
Blood-based toxicity markers for all patients as a function of time since start of therapy

Blood toxicity markers recorded starting from baseline (1–2 weeks before the start of cycle 1) and ending with the most recent follow-up available for each patient are shown in Fig. 5. Considering all follow-up collected so far, 8/20 patients experienced at least one new grade 3 + toxicity in one of these toxicity markers with 2 thrombocytopenia, 2 neutropenia, 5 lymphopenia, 1 leukopenia, and 2 anemia. 3 patients had more than one grade 3 + toxicity.

### Correlation between dosimetry and blood values

**Fig. 6:**
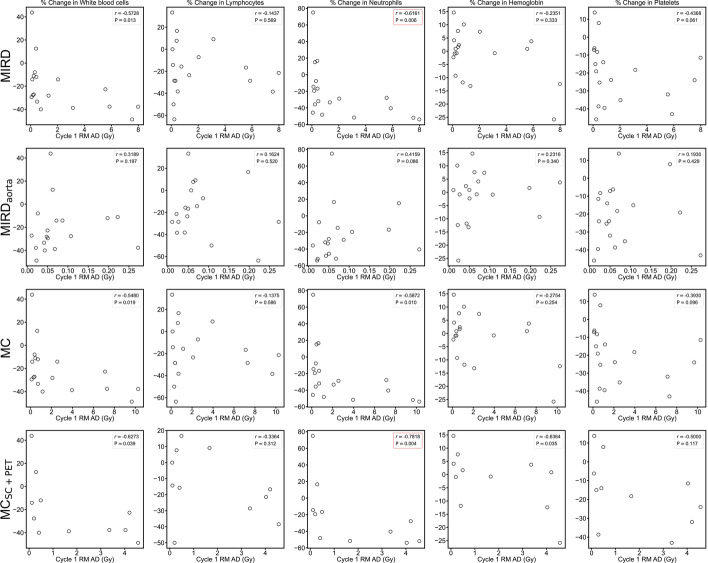
Spearman rank correlation coefficient and significance for cycle 1 AD (Gy) calculated with each dosimetry method correlated with % change in blood toxicity markers following cycle 1 of therapy at approximately 6 weeks. Significant (P ≤ 0.01, with Bonferroni correction) correlations are indicated by a red outline around the legend

Significant correlations between change in some baseline to post-cycle 1 blood values and cycle 1 AD to RM were observed for MIRD and MC_SC+PET_, but not MC or MIRD_aorta_ (Fig. 6). For patients that had follow-up recorded at approximately 6 months following the start of therapy (*N* = 15), longer-term changes in white blood cell count were also correlated with cycle 1 RM AD (Fig. S7 in Online Resource 1) but only for MC_SC+PET_ (*r* = − 0.80, *P* < 0.01). If all methods are restricted to the same subset of 12 patients available for MC_SC+PET_, then MIRD and MC had significant correlations with acute changes in neutrophils (MIRD: *r* = − 0.88, *P *< 0.01; MC: r = − 0.88, *P* < 0.01) and chronic changes in white blood cells (MIRD: *r* = − 0.80, *P* < 0.01; MC: *r* = − 0.80, *P* < 0.01), similar to MC_SC+PET_. MIRD_aorta_ remained uncorrelated with changes in blood values (Fig. S8–S9 in Online Resource 1). Patient P12 is excluded from all correlation analyses due to an active infection potentially affecting blood counts at baseline.

## Discussion

This work compares RM ADs computed with 4 different image-based dosimetry techniques. The MIRD_aorta_ RM ADs were not concordant with the ADs computed with MIRD, MC, and MC_SC+PET_ and likely represent an underestimation of the RM AD. With MC_SC+PET_, the newly-proposed method that uses information on the patient specific marrow and tumor distribution in the spongiosa, ADs were notably lower among patients with large bone tumor load compared to MIRD (39%) or MC (53%), although still higher than MIRD_aorta_. RM ADs for MIRD, MC, and MC_SC+PET_ correlated significantly with the relative percent change in certain blood toxicity markers (white blood cells and neutrophils) considering both acute (6 weeks post-cycle 1) and chronic (6 months post-cycle 1) changes. No correlations for MIRD_aorta_ were significant.

Some previous reports for BM dosimetry that rely on blood-based dosimetry and/or blood-based dosimetry with non-self-dose computed from planar imaging report mean ADs ranging from 0.012–0.034 Gy/GBq [3–6]. These ADs agree well with MIRD_aorta_ ADs (0.01 ± 0.01 Gy/GBq), but our other image-based methodologies trend higher with mean normalized ADs ranging from 0.24 to 0.36 Gy/GBq. This is not unexpected since the MIRD_aorta_ calculation is agnostic to the patient-specific differences in healthy marrow and tumor distributions and we might reasonably expect that these ADs are underestimates of the true value. In some cases, using a blood-based surrogate is a reasonable approach, particularly when there is no specific uptake in bone marrow or no macroscopic infiltration of tumor in the spongiosa. However, since it does not account for cross-dose and changes to the marrow distribution when tumor infiltration is present, such an approach is less suitable for mCRPC patients with extensive bone disease, even though there is no specific uptake of [^177^Lu]Lu-PSMA-617 in bone marrow. Furthermore, the RMBLR chosen for MIRD_aorta_ dosimetry is the HCT-based formula provided by Sgouros [24] with a red marrow extracellular fluid fraction of 0.19 (Eq. 2). This was originally derived for ^131^I-labelled monoclonal antibodies, particles that are much larger than [^177^Lu]Lu-PSMA-617. It has been demonstrated that a RMBLR of 1 is appropriate for [^177^Lu]Lu-DOTA-TATE [36] and some recommendations in the literature indicate that this result should be translated to [^177^Lu]Lu-PSMA-617 [37]. The lower RMBLR (average 0.3) calculated with the HCT-based formula was used based on some prior reports of blood-based marrow dosimetry in [^177^Lu]Lu-PSMA-617 that used RMBLR < 1 [6, 8], but some recent reports do adopt RMBLR = 1 [38]. However, even assuming the same initial distribution volume as [^177^Lu]Lu-DOTA-TATE due to particle size, we would expect the RMBLR to be lower for [^177^Lu]Lu-PSMA-617, because the measured RMBLR for [^177^Lu]Lu-DOTA-TATE would necessarily include the activity contribution from specific binding and internalization of tracer mediated by SSTR receptors expressed on bone marrow cells (specifically, CD34( +) hematopoietic progenitor cells [39]), which is not expected for [^177^Lu]Lu-PSMA-617 as PSMA receptors are not expressed in bone marrow [2]. In our study, using a RMBLR of 1 instead of the formula of Sgouros increases the ADs computed with MIRD_aorta_ by a factor ~ 3, but the main interpretation of the data does not change. In cases with significant bone metastases, ADs are much higher for image-based methodologies than for blood-based (Fig. 4 and Fig. S4), and significant correlations with blood toxicity markers disappear, indicating the importance of image-based marrow dosimetry for these patients.

When comparing patients with large tumor loads (who tend to have high RM AD) across our other three methods, AD is lower with MC_SC+PET_ than either MIRD or MC. We found that the average ADs for 3 patients with a high bone metastatic burden are 39% lower than MIRD and 53% lower than MC ADs (Fig. 4 and Fig. S4). This is likely due to the localization of tumor and marrow with [^68^Ga]Ga-PSMA-11 PET/CT and [^99m^Tc]Tc-sulfur colloid SPECT/CT, respectively. Only self-dose was considered with the MIRD image-based dosimetry methodology, and despite missing AD from cross-dose from other organs and the rest of body, AD estimates from this method were still generally higher than with MC_SC+PET_, especially in patients with larger tumor loads. This reliance on self-dose does, however, partly explain the systematically lower MIRD ADs when compared to the image-based MC methodology which implicitly takes into account cross-dose from the entire field-of-view (Fig. 4 and Fig. S4).

Without any information about the marrow distribution of each patient, only standard models of marrow distributions can be used, and they may not accurately reflect the distribution in the patient, especially for patients with large amounts of tumor infiltration in the spongiosa as the impinging disease may result in displacement of RM. In some prior studies of [^177^Lu]Lu-DOTA-TATE PRRT [40, 41], ADs are calculated considering only the counts in spongiosa regions where tumor encroachment is not evident (uniform/no focal uptake), however this can lead to underestimation of the skeletal average AD, especially in ^177^Lu RLT where high skeletal tumor burden is common. In contrast, including the entire spongiosa without differentiating tumor and marrow as was the case with MIRD and MC methods in the current study, will lead to overestimation due to over assigning activity to the RM that should have been attributed to tumor. From the literature, image-based PSMA dosimetry methodologies tend to report higher ADs than blood-based methodologies. For example, the median AD and range was 0.10 Gy/GBq (0.01–0.34 Gy/GBq) from Violet et al. [9] and 0.130 Gy/GBq (0.004–0.933 Gy/GBq) from Gosewisch et al. [8] Alternatively, ignoring pathologically-involved skeletal sites and restricting AD calculation to regions that are apparently healthy may result in underestimation of the AD because some RM may be receiving increased cross-dose from encroaching tumor in the ignored regions. For example, an alternative calculation from Gosewisch et al. assumes complete displacement from sites of tumor (unlike MIRD and MC in this work, which do not attempt to localize tumor) and reports 0.037 Gy/GBq (0.004–0.106 Gy/GBq) [8], more closely matching blood-based approaches. It is noteworthy that Gosewisch et al. additionally report AD for a calculation methodology which uses [^99m^Tc]Tc-anti-granulocyte antibody SPECT/CT for marrow localization, however, it is reported only for two cases and does not employ micro-scale dosimetry models for co-localized marrow and tumor. For the two patients with [^99m^Tc]Tc-anti-granulocyte antibody-informed dosimetry, their ADs fell between the other two MC methods (i.e. with and without complete tumor displacement) [8]. A recent work from Grob et al. in hormone sensitive patients receiving [^177^Lu]Lu-PSMA-617 also looked at the differences between blood-based and image-based dosimetry and also found that image-based ADs were calculated to be higher than blood-based [42].

This work is only pertinent for clinical application of [^177^Lu]Lu-PSMA RLT if hematological toxicities are a concern in these patients. From the literature we see that the most common grade 3+ toxicities are hematological: 12.9% anemia, 7.9% thrombocytopenia, 7.8% lymphopenia, and 2.5% leukopenia from 529 patients on the phase-III VISION trial [13] and 11% thrombocytopenia, 8% anemia, 4% neutropenia, and 1% leukopenia from 98 patients on the phase-II TheraP trial [12]. As such, understanding the dose-toxicity relationship for these patients can play a critical role in limiting these toxicities and personalizing ^177^Lu RLT. Eight out of twenty study patients experienced at least one new grade 3 + hematological toxicity over the course of their treatment or in follow-up, including 3 patients with more than one grade 3 + toxicity. However, this relatively high incidence is potentially biased for our population due to a few factors. First, we considered a longer follow-up period for evaluating toxicity than previous clinical trials, with follow-up for some patients available more than a year since the start of therapy. Second, the patients initially treated at our institution were the ones most in need of the therapy. Lastly, patients with extensive bone disease and/or depleted marrow reserves were more likely to be recommended to receive clinical dosimetry. The mean ADs for our image-based dosimetry methods are near the commonly cited 2 Gy toxicity threshold used to guide therapy of differentiated thyroid cancer with radioiodine [43]. However, this mean is across dichotomous groups of patients, those who were much lower than the 2 Gy limit and others who exceed it, with the mean dominated by the latter (Fig. 4). Furthermore, these limits are defined with blood as a surrogate, and might not be applicable in general, and to [^177^Lu]Lu-PSMA RLT in particular, as indicated in the current work. Direct comparison of AD from different radiopharmaceuticals must be done with caution, as differences in radiobiology due to differences in dose-rate associated with different half-lives, decay products, and molecule distribution/binding mechanics can result in differences in dose-toxicity relationships. Lastly, the numerical values are strongly method dependent, as different assumptions and model parameters are used.

For MC_SC+PET_, we used the [^99m^Tc]Tc-sulfur colloid imaging to derive patient-specific BM distributions, but due to availability of the special imaging study, this method was restricted to only 12 patients. The RM quantification is dependent on a population-based cellularity assumption in the reference region. In some patients, this assumption is likely not valid due to widespread metastases affecting all marrow-bearing regions. In these cases, a solution may be to use a population-level average for sulfur-colloid uptake per marrow mass, but larger sample sizes of patients with acceptable reference regions need to be acquired before such an average can be established. Another consideration with such an approach is that the [^99m^Tc]Tc-sulfur colloid radiopharmaceutical was prepared in-house and the manufacturing process creates different proportions of particle sizes in the final mixture with only the smallest particles localizing to macrophages associated with active marrow [44]. So, while our patient-specific scaling of [^99m^Tc]Tc-sulfur colloid activity concentration overcomes this limitation, comparing scale factors across patients may not be possible. Also note [^99m^Tc]Tc-sulfur colloid localizes to reticuloendothelial cells which may not necessarily be a perfect surrogate for stimulated RM. This also means most of the uptake in the patient is in the liver and spleen, which may lead to spill-in of activity to the marrow space, particularly for the superior lumbar and inferior thoracic vertebrae, although our images are reconstructed quantitatively with scatter correction, so this effect should be minimal [31]. Alternative methods for patient-specific cellularity determination such as using MRI [45], dual energy CT [46], [^18^F]FLT PET [47], or imaging with other ^99m^Tc -labelled tracers [8] could also be explored.

While our RM AD estimates consider patient-specific differences in tumor and RM distributions, there are still limitations to our approach. Our micro-scale MC modeling of the spongiosa with varying CF and BVF does not perfectly reflect the biological reality of the trabecular space. Modeling of additional features including clustering of YM subvoxels, adjusted distribution of RM and YM based on proximity to the bone surface, and a blood vessel compartment to contain circulating activity will be considered in future iterations of this workflow to further enhance the computational accuracy of AD to RM. The impact of partial volume effects on activity quantification accuracy due to poor spatial resolution, especially in the case of SPECT, is well known [48]. The associated activity spill-out makes quantifying activity in low-uptake BM regions adjacent to high uptake tumor especially challenging. We mitigated this effect by leveraging the activity in the aorta as an indirect measure of what the BM activity should be and comparing with what is measured in the spongiosa, assigning excess activity back into tumor, as necessary. However, the aorta is not exempt from partial volume effects and activity quantification of the structure may also be affected by nearby high activity regions as well as the long, narrow shape of the aorta. Other challenges include the difficulty of spongiosa segmentation. We leveraged open-source deep learning segmentation models with custom post-processing to generate spongiosa segmentations, but alternative strategies exist, such as using small spherical VOIs in the center of each marrow-bearing region [20]. However, such approaches are not applicable when trying to account for patient-specific tumor and marrow distributions like with MC_SC+PET_. Tumor segmentation was only attempted for MC_SC+PET_, but also presents challenges, particularly in quantifying the level of tumor involvement in regions of overlap between segmented tumor and localization of RM. We assumed the volume of marrow and tumor were equal when both tumor and RM were colocalized, but other ways of determining tumor involvement of marrow on the subvoxel level should be explored, potentially based on PSMA uptake intensity. The quantification and localization of tumor and marrow facilitated by additional coregistered [^68^Ga]Ga-PSMA-11 PET/CT and ^99m^Tc-SC SPECT/CT, respectively, are highly dependent on the coregistration technique. Deformable image registration was employed to transform the CT of each series to the reference CT of the ^177^Lu SPECT/CTs, but accurate registration of the 3 images is still challenging and contributes to the variability and uncertainty of the dosimetry estimates as does the multiple local rigid registration technique used to coregister the multi-time point ^177^Lu SPECT/CT images for TIA map generation. The registration of multi-time point ^177^Lu SPECT/CT images was confirmed visually with particular attention paid to marrow-bearing regions. The TIA quantification accuracy has been quantified for tumors and kidney [49], and the method has been used by our group previously for voxel-level dosimetry [50]. While voxel-level dosimetry may introduce some noise into individual fits, as shown previously, TIA estimates and fits are still robust [50]. Furthermore, voxel-level fitting in this case is necessary to allow for sub-organ level investigation of AD. The timing of the ^99m^Tc-SC SPECT/CT relative to therapy may also play a role in the accuracy of using the scan for evaluating patient-specific RM distributions. Ideally, the acquisition should be taken immediately before the start of the first therapy cycle, but due to the clinical nature of the scans utilized in this study, this was usually not the case. Future work should try to standardize the ^99m^Tc-SC SPECT/CT timing to the week prior to therapy in order to most accurately capture the RM distribution at administration and mitigate the potential effects of [^177^Lu]Lu-PSMA-617 itself, tumor progression, or therapeutic response following the first cycle.

The dose-toxicity correlations are limited by the blood sampling schedule for measuring blood toxicity markers. Most patients only had blood draws approximately 6 weeks after each cycle while the nadir for blood counts, which would more accurately capture the acute toxicity response of the therapy, is typically 3–6 weeks post-therapy [51]. Additionally, the number of patients recruited to the study is limited due to the imaging requirements, hence the dose – toxicity findings need to be validated in a larger cohort. A larger analysis is made more feasible by the semi-automatic nature of many of the processing steps including spongiosa segmentation and deformable image registration. Four of the patients in this study had treatment halted due to hematological toxicity, indicating the need for additional work on establishing predictors of such toxicity.

## Conclusion

We applied image-based dosimetry via the MIRD schema as well as 3D MC dosimetry with micro-scale spongiosa modeling to patients undergoing [^177^Lu]Lu-PSMA-617 RLT for the treatment of mCRPC. We found the blood-based dosimetry surrogate was not concordant with other image-based AD estimates and underestimates AD, particularly in patients with bone metastases. Furthermore, among the remaining image-based methodologies, AD to patients is estimated to be lower when utilizing the additional [^68^Ga]Ga-PSMA-11 PET/CT and [^99m^Tc]Tc-sulfur colloid SPECT/CT imaging studies to localize RM and tumor. Significant correlations between imaging-based dosimetry results and blood counts were found which were not present with blood-based MIRD calculations. These results indicate the necessity for image-based dosimetry studies in [^177^Lu]Lu-PSMA-617 RLT for establishing robust dose-toxicity relationships with the ultimate goal of pre-therapy/cycle toxicity prediction for patient selection and management.

## Supplementary Information

Additional file1 (PDF 2479 KB)

Additional file2 (XLSX 123 KB)

Additional file3 (PDF 39 KB)

Additional file4 (PDF 61 KB)

## Abbreviations

AD: Absorbed dose
BM: Bone marrow
BVF: Bone volume fraction
CCC: Concordance correlation coefficient
CF: Cellularity fraction
CT: Computed tomography
CTCAE: Common terminology criteria for adverse events
DPM: Dose planning method
FOV: Field of view
HCT: Hematocrit
ICRP: International commission on radiological protection
MC: Monte Carlo
mCRPC: Metastatic castrate resistant prostate cancer
MIRD: Medical internal radiation dose
MRI: Magnetic resonance imaging
PET: Positron emission tomography
PRRT: Peptide receptor radionuclide therapy
PSMA: Prostate specific membrane antigen
RLT: Radioligand therapy
RM: Red marrow
RMBLR: Red marrow to blood activity concentration ratio
SC: Sulfur colloid
SPECT: Single photon emission computed tomography
TAC: Time-activity curve
TIA: Time-integrated activity
VOI: Volume of interest
YM: Yellow marrow

## Declarations

### Availability of data and materials

The datasets generated during and/or analyzed during the current study are available from the corresponding author on reasonable request.
