# Supplementary material for: Comparison of imaging-based bone marrow dosimetry methodologies and their dose–effect relationships in [177Lu]Lu-PSMA-617 RLT including a novel method with active marrow localization
Source: EJNMMI Phys. 2025 Dec 4;13:1. doi: 10.1186/s40658-025-00816-6 (PMC12779781; doi:10.1186/s40658-025-00816-6)
Supplement: Supplementary file 1 — Additional file1 (PDF 2479 KB) [file 40658_2025_816_MOESM1_ESM.pdf]

ONLINE RESOURCE 1 – SUPPLEMENTAL TABLES AND FIGURES

Supplemental Table 1. Patient characteristics at baseline

Patient	Age (y)	# Completed Cycles	Total Injected Activity (GBq)	Gleason Score	Baseline PSA (ng/mL)	Baseline White Blood Cells (×10³/mL)	Baseline Absolute Lymphocytes (×10³/mL)	Baseline Absolute Neutrophils (×10³/mL)	Baseline Hemoglobin (g/dL)	Baseline Platelets (×10³/mL)	Previous Systemic Therapy (Y/N)
**P1**	79	4	28.7	7	175	8.5	2.1	5.6	9.3	357	Y
**P2**	77	5	34.8	7	602	9.7	1.2	7.9	11.4	247	Y
**P3**	70	6	44.0	9	20	8	1.1	6.2	13.2	241	Y
**P4**	69	4	28.9	7	21	9.2	1.4	6.9	12.2	218	Y
**P5**	64	4	28.8	9	8	4.8	1.3	2.8	12.3	177	Y
**P6**	68	4	28.9	6	430	4.7	0.8	3.1	11.2	246	Y
**P7**	66	2	13.5	9	1575	7.8	1.3	5.4	12.4	204	Y
**P8**	74	1	5.8	7	423	4.5	1.4	2.6	12.1	139	Y
**P9**	78	6	43.4	9	49	6.9	1.4	4.7	10.7	286	Y
**P10**	66	6	43.4	9	72	7.5	0.6	6	12.1	241	Y
**P11**	63	6	42.9		27	5.6	1.3	3.6	11.8	357	Y
**P12**	77	5	36.1	9	10						Y
**P13**	56	1	7.2	7	328	10.8	0.6	9.4	8.9	167	Y
**P14**	70	6	38.3	10	46	8.5	3.2	3.61	9.5	398	Y
**P15**	78	2	14.0	8	316	7.1	1.7	4.5	11.4	184	Y
**P16**	71	4	28.1		134	4.5	1.1	2.692	14	309	Y
**P17**	74	2	14.8		97	5.1	0.6	3.5	13	183	Y
**P18**	58	2	14.5		13	4.4	1.4	2.6	12.6	206	Y
**P19**	76	1	7.5		217	7	0.7	5.6	11.7	172	Y
**P20**	65	2	12.0		62	4.8	1.41	2.76	12.7	284	Y

Supplemental Table 2. Imaging acquisition and reconstruction parameters

	^{177}Lu SPECT				^{177}Lu CT		^{68}Ga PET				^{99m}Tc-sulfur colloid SPECT			
Patient	Scanner	Matrix Size	Voxel Size	Reconstruction	Matrix Size	Voxel Size	Scanner	Matrix Size	Voxel Size	Reconstruction	Scanner	Matrix Size	Voxel Size	Reconstruction
**P1**	Siemens Intevo Bold	256× 256	1.95mm× 1.95mm× 1.95mm	xSPECT, OSCGMM 48i1s, no post-filtering	512× 512	0.98mm× 0.98mm× 3mm	Siemens Biograph mCT	200× 200	4.07mm× 4.07mm× 2mm	PSF+TOF 3i21s, 4mm Gaussian post-filtering	Siemens Intevo Bold	256× 256	1.95mm× 1.95mm× 1.95mm	xSPECT, OSCGMM 48i1s, no post-filtering
**P2**	Siemens Intevo Bold	256× 256	1.95mm× 1.95mm× 1.95mm	xSPECT, OSCGMM 48i1s, no post-filtering	512× 512	0.98mm× 0.98mm× 1.5mm	Siemens Biograph mCT	200× 200	4.07mm× 4.07mm× 2mm	PSF+TOF 3i21s, 4mm Gaussian post-filtering	Siemens Intevo Bold	256× 256	1.95mm× 1.95mm× 1.95mm	xSPECT, OSCGMM 48i1s, no post-filtering
**P3**	Siemens Intevo Bold	256× 256	1.95mm× 1.95mm× 1.95mm	xSPECT, OSCGMM 48i1s, no post-filtering	512× 512	0.98mm× 0.98mm× 1.5mm	Siemens Biograph mCT	200× 200	4.07mm× 4.07mm× 2mm	PSF+TOF 3i21s, 4mm Gaussian post-filtering	Siemens Intevo Bold	256× 256	1.95mm× 1.95mm× 1.95mm	xSPECT, OSCGMM 48i1s, no post-filtering
**P4**	Siemens Intevo Bold	256× 256	1.95mm× 1.95mm× 1.95mm	xSPECT, OSCGMM 48i1s, no post-filtering	512× 512	0.98mm× 0.98mm× 1.5mm	Siemens Biograph mCT	200× 200	4.07mm× 4.07mm× 2mm	PSF+TOF 3i21s, 4mm Gaussian post-filtering	Siemens Intevo Bold	256× 256	1.95mm× 1.95mm× 1.95mm	xSPECT, OSCGMM 48i1s, no post-filtering
**P5**	Siemens Intevo Bold	256× 256	1.95mm× 1.95mm× 1.95mm	xSPECT, OSCGMM 48i1s, no post-filtering	512× 512	0.98mm× 0.98mm× 1.5mm	Siemens Biograph TruePoint	168× 168	4.07mm× 4.07mm× 3mm	PSF 3i21s, 4mm Gaussian post-filtering	Siemens Intevo Bold	256× 256	1.95mm× 1.95mm× 1.95mm	xSPECT, OSCGMM 48i1s, no post-filtering
**P6**	Siemens Intevo Bold	256× 256	1.95mm× 1.95mm× 1.95mm	xSPECT, OSCGMM 48i1s, no post-filtering	512× 512	0.98mm× 0.98mm× 1.5mm	GE Discovery STE	128× 128	5.47mm× 5.47mm× 3.27mm	3D IR	Siemens Intevo Bold	256× 256	1.95mm× 1.95mm× 1.95mm	xSPECT, OSCGMM 48i1s, no post-filtering
**P7**	Siemens Intevo Bold	256× 256	1.95mm× 1.95mm× 1.95mm	xSPECT, OSCGMM 48i1s, no post-filtering	512× 512	0.98mm× 0.98mm× 1.5mm	Siemens Biograph mCT	200× 200	4.07mm× 4.07mm× 2mm	PSF+TOF 3i21s, 4mm Gaussian post-filtering	Siemens Intevo Bold	256× 256	1.95mm× 1.95mm× 1.95mm	xSPECT, OSCGMM 48i1s, no post-filtering
**P8**	Siemens Intevo Bold	256× 256	1.95mm× 1.95mm× 1.95mm	xSPECT, OSCGMM 48i1s, no post-filtering	512× 512	0.98mm× 0.98mm× 1.5mm	Siemens Biograph mCT	200× 200	4.07mm× 4.07mm× 2mm	PSF+TOF 3i21s, 4mm Gaussian post-filtering	Siemens Intevo Bold	256× 256	1.95mm× 1.95mm× 1.95mm	xSPECT, OSCGMM 48i1s, no post-filtering
**P9**	Siemens Intevo Bold	256× 256	1.95mm× 1.95mm× 1.95mm	xSPECT, OSCGMM 48i1s, no post-filtering	512× 512	0.98mm× 0.98mm× 1.5mm	Siemens Biograph mCT	400× 400	2.04mm× 2.04mm× 3mm	PSF 2i24s, 3mm Gaussian post-filtering	Siemens Intevo Bold	256× 256	1.95mm× 1.95mm× 1.95mm	xSPECT, OSCGMM 48i1s, no post-filtering
**P10**	Siemens Intevo Bold	256× 256	1.95mm× 1.95mm× 1.95mm	xSPECT, OSCGMM 48i1s, no post-filtering	512× 512	0.98mm× 0.98mm× 1.5mm	Siemens Biograph mCT	200× 200	4.07mm× 4.07mm× 2mm	PSF+TOF 3i21s, 4mm Gaussian post-filtering	Siemens Intevo Bold	256× 256	1.95mm× 1.95mm× 1.95mm	xSPECT, OSCGMM 48i1s, no post-filtering
**P11**	Siemens Intevo Bold	256× 256	1.95mm× 1.95mm× 1.95mm	xSPECT, OSCGMM 48i1s, no post-filtering	512× 512	0.98mm× 0.98mm× 1.5mm	Siemens Biograph mCT	200× 200	4.07mm× 4.07mm× 2mm	PSF+TOF 3i21s, 4mm Gaussian post-filtering	Siemens Intevo Bold	256× 256	1.95mm× 1.95mm× 1.95mm	xSPECT, OSCGMM 48i1s, no post-filtering
**P12**	Siemens Intevo Bold	256× 256	1.95mm× 1.95mm× 1.95mm	xSPECT, OSCGMM 48i1s, no post-filtering	512× 512	0.98mm× 0.98mm× 1.5mm	Siemens Biograph mCT	200× 200	4.07mm× 4.07mm× 2mm	PSF+TOF 3i21s, 4mm Gaussian post-filtering	Siemens Intevo Bold	256× 256	1.95mm× 1.95mm× 1.95mm	xSPECT, OSCGMM 48i1s, no post-filtering
**P13**	Siemens Intevo Bold	256× 256	1.95mm× 1.95mm× 1.95mm	xSPECT, OSCGMM 48i1s, no post-filtering	512× 512	0.98mm× 0.98mm× 1.5mm								
**P14**	Siemens Intevo Bold	256× 256	1.95mm× 1.95mm× 1.95mm	xSPECT, OSCGMM 48i1s, no post-filtering	512× 512	0.98mm× 0.98mm× 1.5mm								
**P15**	Siemens Intevo Bold	256× 256	1.95mm× 1.95mm× 1.95mm	xSPECT, OSCGMM 48i1s, no post-filtering	512× 512	0.98mm× 0.98mm× 1.5mm								
**P16**	Siemens Intevo Bold	256× 256	1.95mm× 1.95mm× 1.95mm	xSPECT, OSCGMM 48i1s, no post-filtering	512× 512	0.98mm× 0.98mm× 1.5mm								
**P17**	Siemens Intevo Bold	256× 256	1.95mm× 1.95mm× 1.95mm	xSPECT, OSCGMM 48i1s, no post-filtering	512× 512	0.98mm× 0.98mm× 1.5mm								

**P18**	Siemens Intevo Bold	256× 256	1.95mm× 1.95mm× 1.95mm	xSPECT, OSCGMM 48i1s, no post-filtering	512× 512	0.98mm× 0.98mm× 1.5mm	
**P19**	Siemens Intevo Bold	256× 256	1.95mm× 1.95mm× 1.95mm	xSPECT, OSCGMM 48i1s, no post-filtering	512× 512	0.98mm× 0.98mm× 1.5mm	
**P20**	Siemens Intevo Bold	256× 256	1.95mm× 1.95mm× 1.95mm	xSPECT, OSCGMM 48i1s, no post-filtering	512× 512	0.98mm× 0.98mm× 1.5mm	

Supplemental Table 3. Detailed steps of spongiosa cleaning workflow as implemented in MIM (MIM Software, Cleveland, OH).

**Step 1**	Subthreshold each skeletal ROI to 400 HU
**Step 2**	Contract contour in anterior-posterior and left-right directions by one voxel
**Step 3**	Morphological opening (left-right and anterior-posterior=2mm, superior-inferior=3mm)
**Step 4**	Morphological closing (left-right and anterior-posterior=2mm, superior-inferior=3mm)
**Step 5**	Smooth contours twice

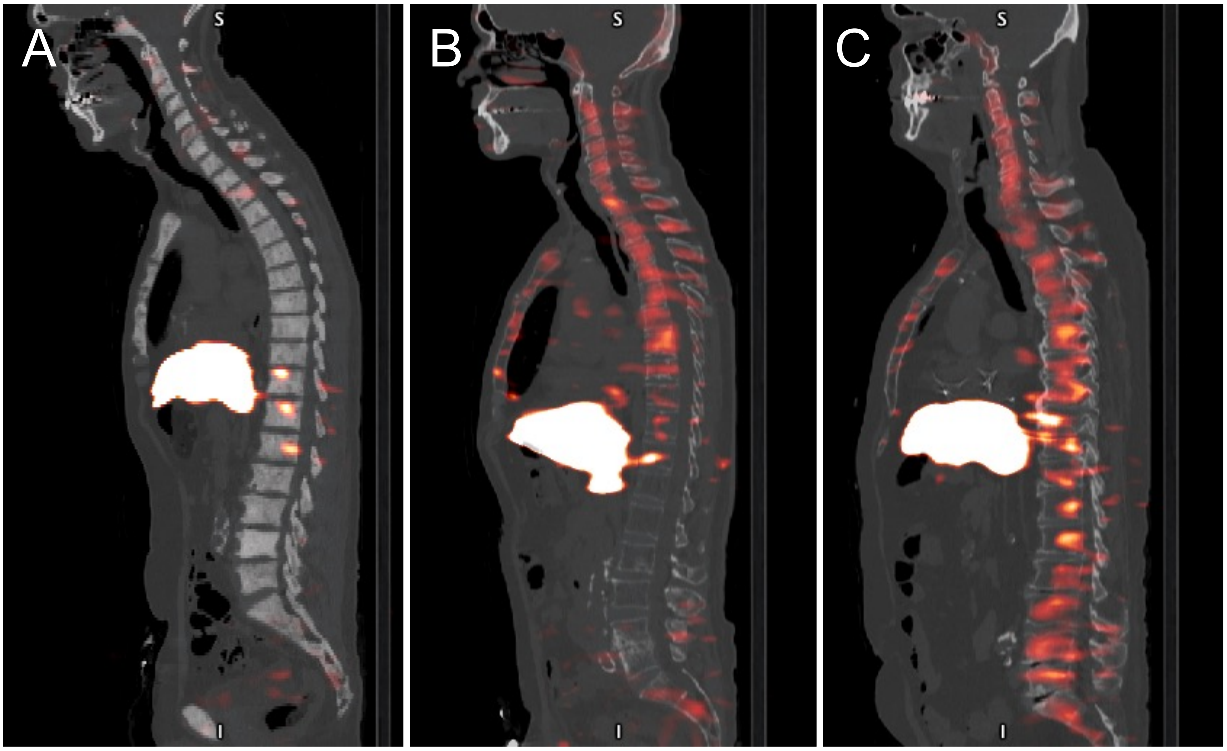

Fig. S1 Sagittal cross-section of [^{99m}Tc]Tc-sulfur colloid SPECT/CT for A) patient P1 indicating low marrow reserves, B) patient P6 indicating mixed marrow reserves, and C) patient P4 indicating relatively high marrow reserves

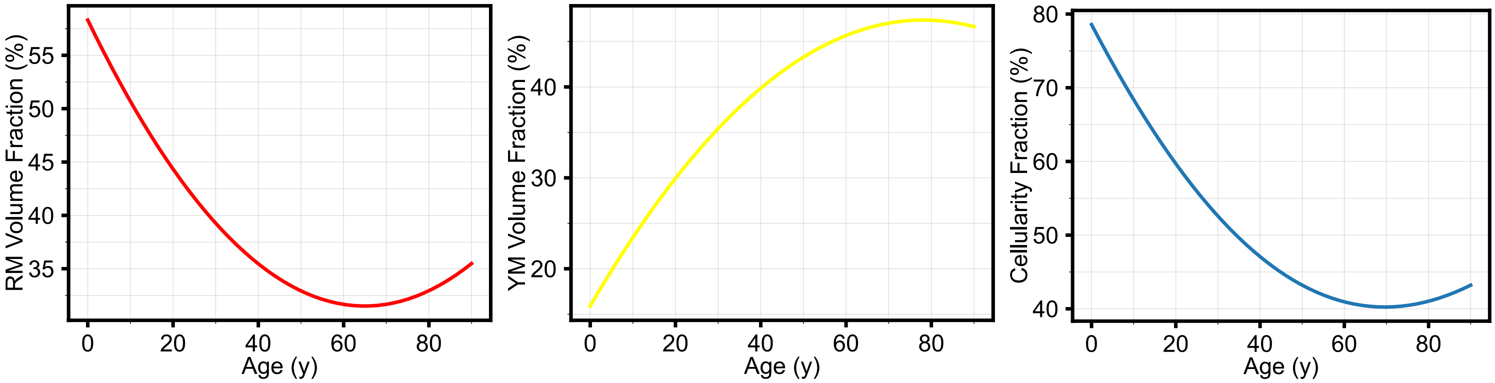

Fig. S2 RM and YM volume fractions as a function of age from Dunnill et al. [31] and the corresponding CF curve as a function of age

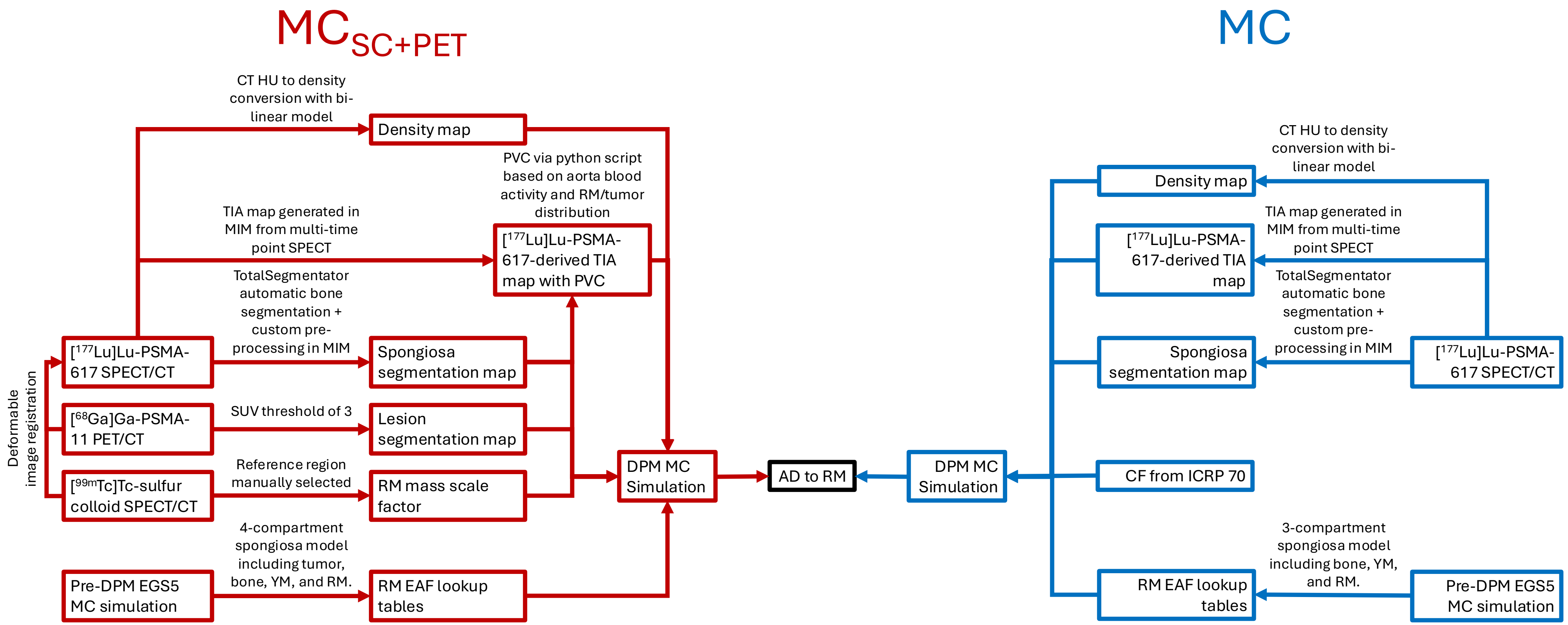

Fig. S3 Flowchart summarizing the MC and MC$_{SC+PET}$ dosimetry methodologies and their inputs to DPM.

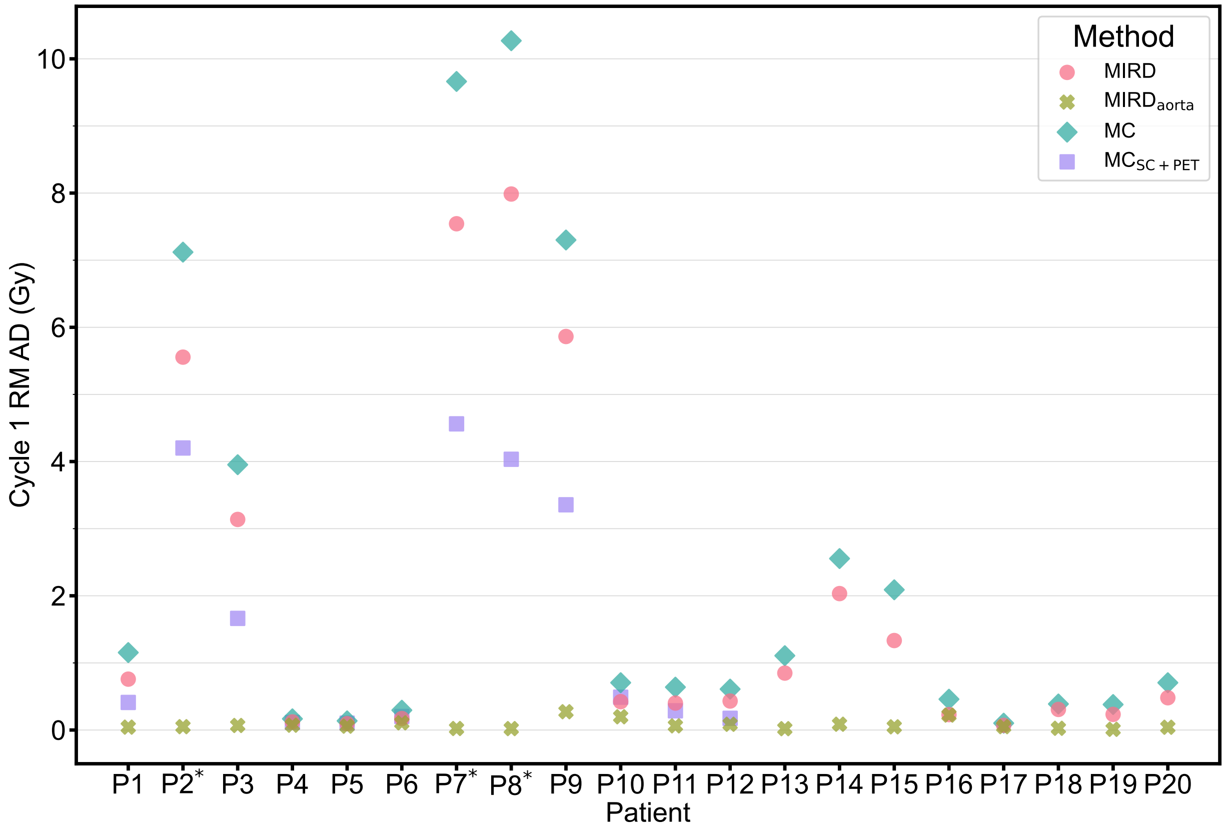

Fig. S4 RM AD for each patient and dosimetry method. Note the lower ADs for MC$_{SC+PET}$, especially in patients with large metastatic burdens (denoted with an asterisk), but also in patients P1, P3, and P9

A

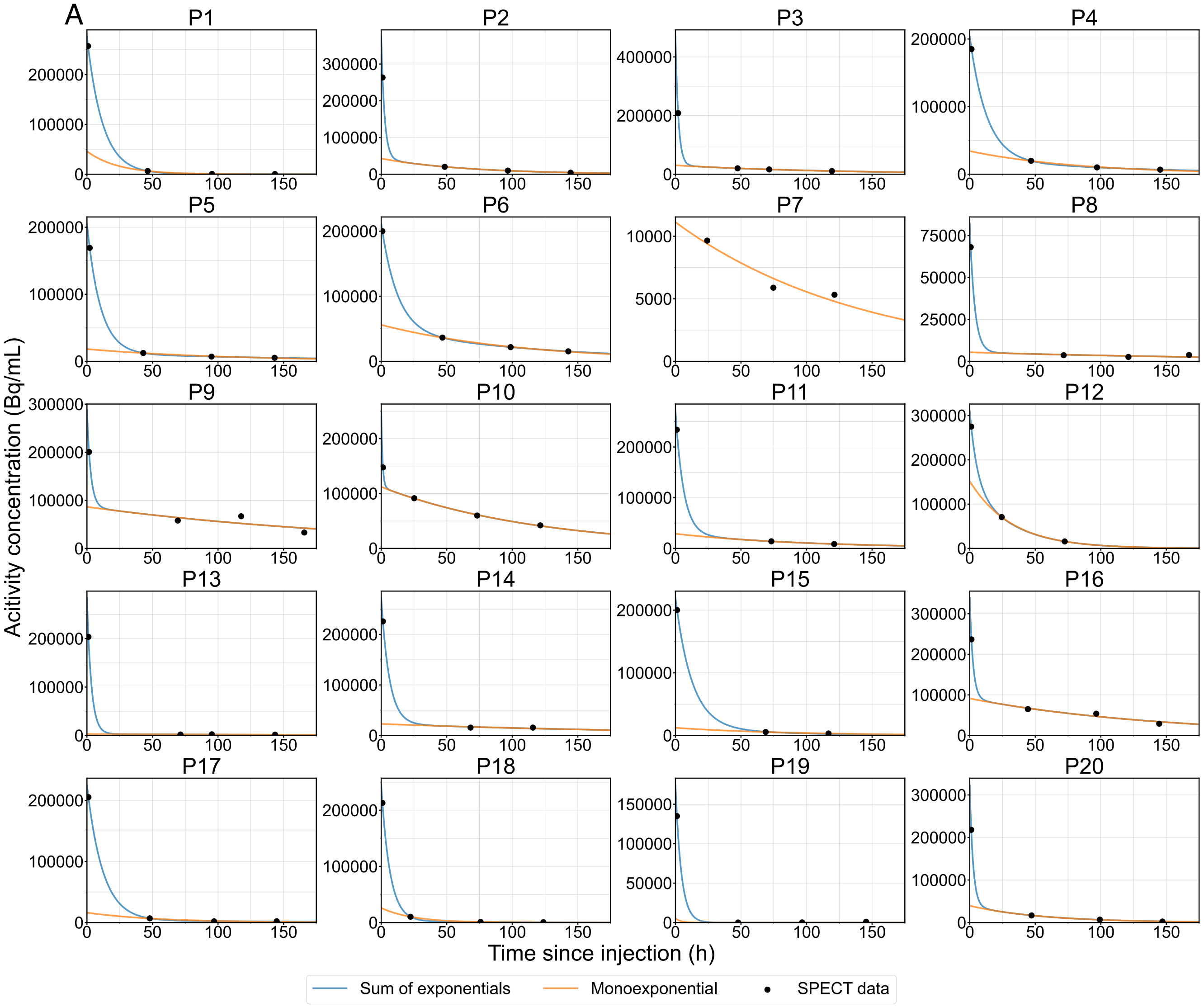

B

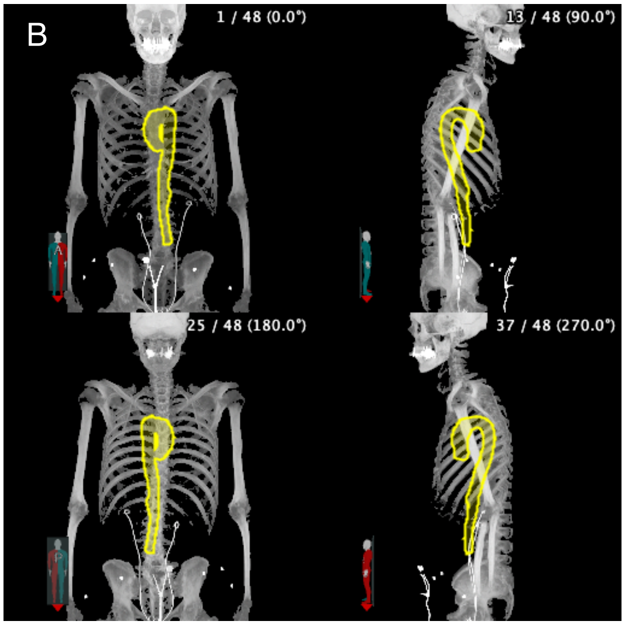

C

Patient	Biexponential T1 (h)	T2 (h)	Monoexponential T (h)
**P1**	8.3	104.8	16.5
**P2**	1.7	45.1	45.1
**P3**	1.6	81.4	81.4
**P4**	9.0	86.4	58.6
**P5**	7.1	117.6	75.0
**P6**	8.5	87.4	74.5
**P7**			99.9
**P8**	2.6	160.0	160.0
**P9**	2.0	160.0	160.0
**P10**	0.7	83.8	83.8
**P11**	4.3	70.0	70.0
**P12**	3.9	22.6	22.2
**P13**	2.3	160.0	160.0
**P14**	4.3	160.0	160.0
**P15**	8.6	42.9	60.3
**P16**	1.7	101.2	101.2
**P17**	8.4	160.0	37.8
**P18**	4.6	54.0	16.8
**P19**	3.1	3.1	2.7
**P20**	1.7	38.2	38.2

Fig. S5 A) Time-activity concentration curves for all 20 patients' aorta activity concentration. Blue fits indicate a bi-phasic exponential fit to the data and the orange curve represents a monoexponential fit to all data >4h post-injection. B) MIP showing aorta contour for patient P2. C) Kinetics of fits to the aorta time-activity concentration data. Note that one patient had only 3 time points with no imaging before 24 h for whom a bi-phasic exponential function could not be fit, so a monoexponential fit of the form: $Cexp(-\lambda t)$ was used instead. In this special case, the $[\tilde{A}]_{aorta}$ is likely underestimated as the blood compartment is known to have a fast, early clearance phase followed by a slower one and the contribution from the early phase would be underestimated

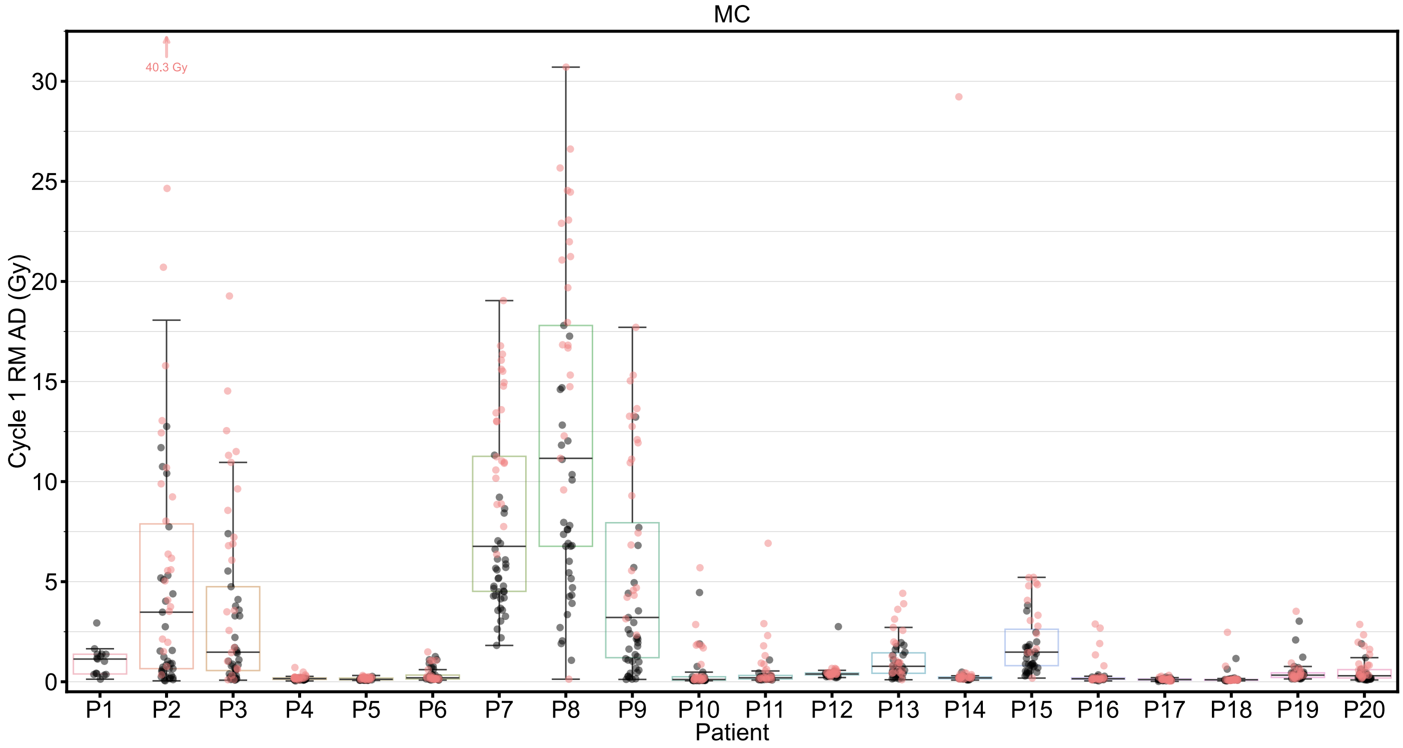

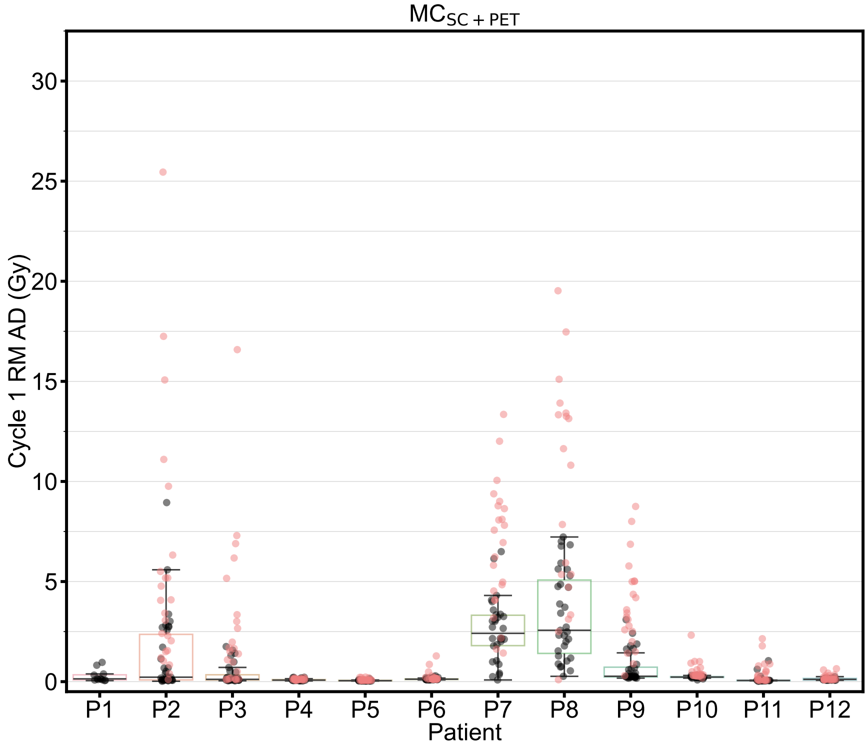

Fig. S6 Box plots displaying AD to each individually segmented ROI that composes the total spongiosa. This is shown for each patient for A) MC and B) MC$_{SC+PET}$ dosimetry methods. AD points for vertebrae are colored red, all other ROIs are black

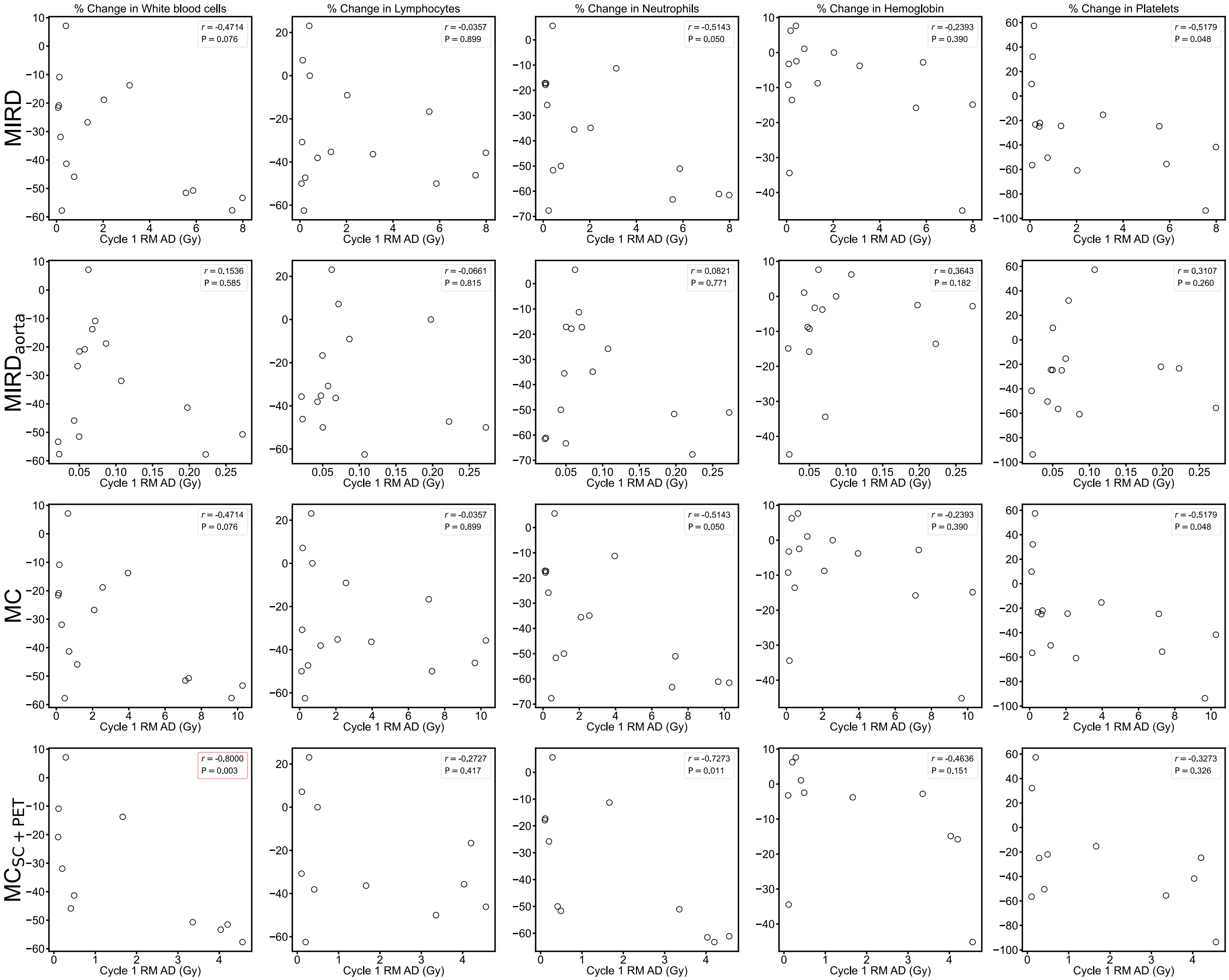

Fig. S7 Spearman rank correlation coefficient and significance for cycle 1 AD (Gy) calculated with each dosimetry method correlated with % change in blood toxicity markers from baseline to approximately 6 months after the start of therapy. Significant (P≤0.01) correlations are indicated by a red outline on the legend

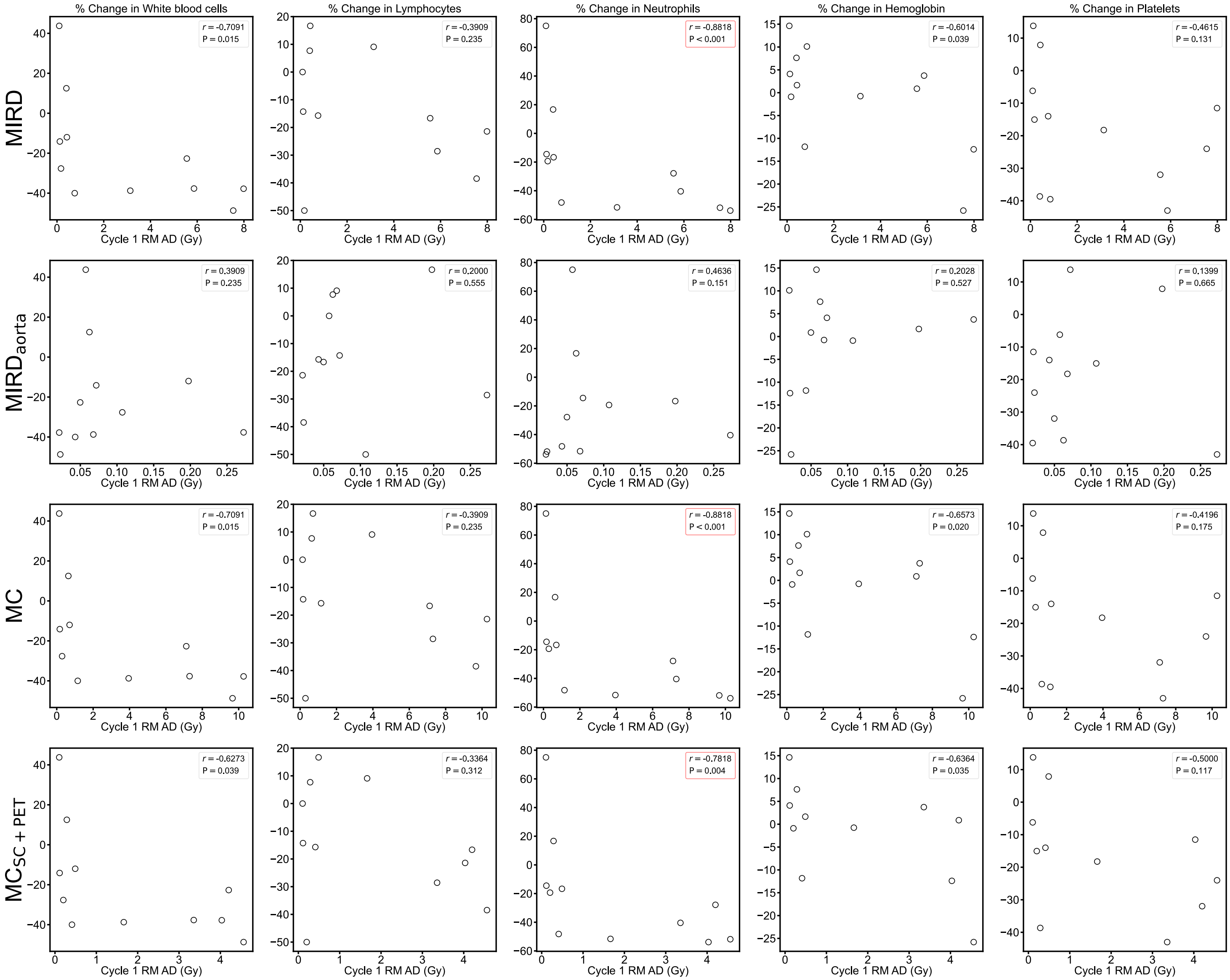

Fig. S8 Spearman rank correlation coefficient and significance for cycle 1 AD (Gy) calculated with each dosimetry method correlated with % change in blood toxicity markers following cycle 1 of therapy at approximately 6 weeks. Significant (P≤0.01, with Bonferroni correction) correlations are indicated by a red outline around the legend. Restricted to P1-P12

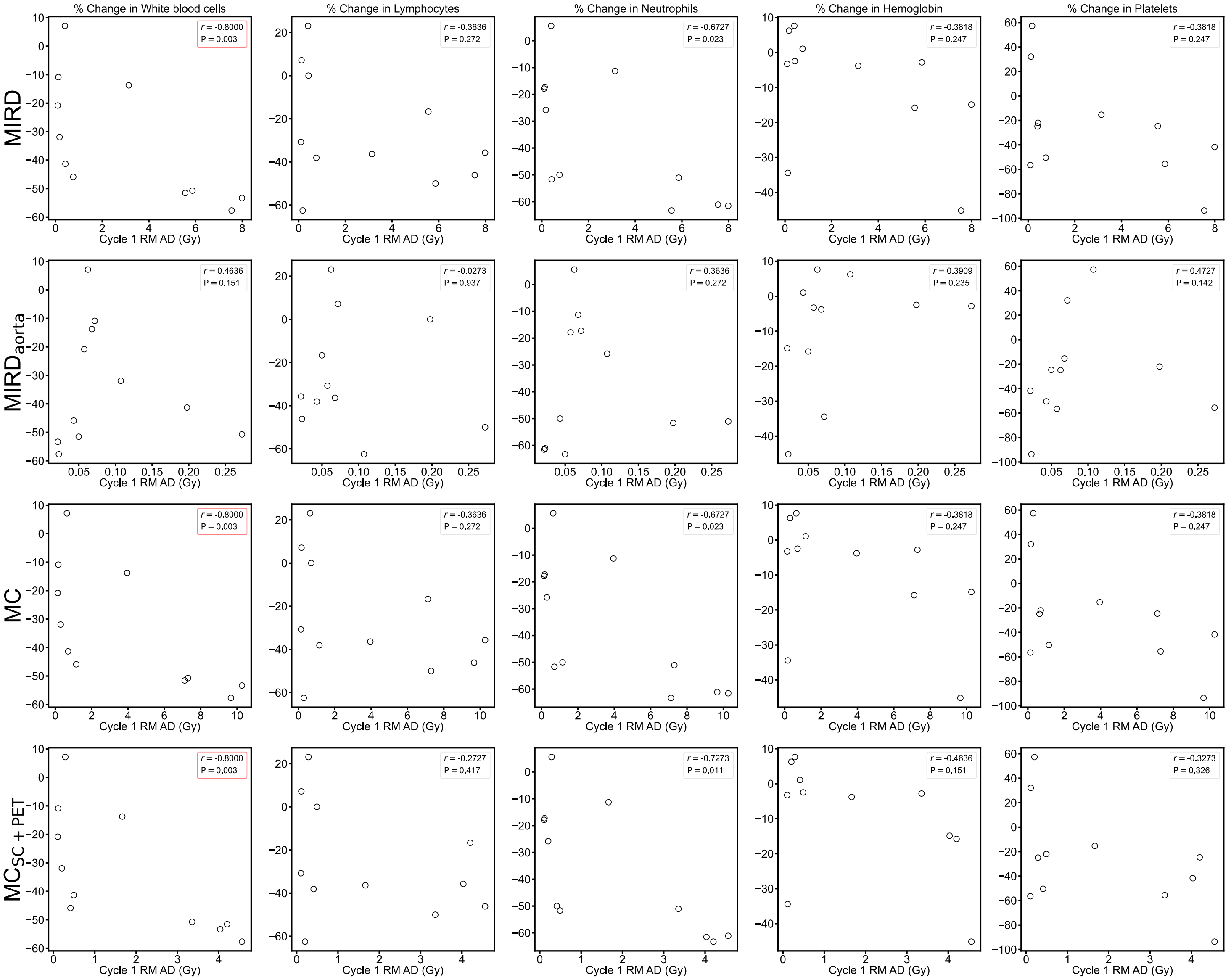

Fig. S9 Spearman rank correlation coefficient and significance for cycle 1 AD (Gy) calculated with each dosimetry method correlated with % change in blood toxicity markers from baseline to approximately 6 months after the start of therapy. Significant (P≤0.01) correlations are indicated by a red outline on the legend. Restricted to P1-P12
