# Supplementary material for: Comparison of imaging-based bone marrow dosimetry methodologies and their dose–effect relationships in [177Lu]Lu-PSMA-617 RLT including a novel method with active marrow localization
Source: EJNMMI Phys. 2025 Dec 4;13:1. doi: 10.1186/s40658-025-00816-6 (PMC12779781; doi:10.1186/s40658-025-00816-6)
Supplement: Supplementary file 3 — Additional file3 (PDF 39 KB) [file 40658_2025_816_MOESM3_ESM.pdf]

ONLINE RESOURCE 3

(FixedInternalImagePixelType "float")

(MovingInternalImagePixelType "float")

(UseDirectionCosines "true")

(Registration "MultiMetricMultiResolutionRegistration")

(Interpolator "BSplineInterpolator")

(ResampleInterpolator "FinalBSplineInterpolator")

(Resampler "DefaultResampler")

(FixedImagePyramid "FixedRecursiveImagePyramid")

(MovingImagePyramid "MovingRecursiveImagePyramid")

(Optimizer "AdaptiveStochasticGradientDescent")

(Transform "BSplineTransform")

(Metric "AdvancedMattesMutualInformation" "TransformBendingEnergyPenalty")

(Metric0Weight 1)

(Metric1Weight 1)

(FinalGridSpacingInVoxels 16)

(GridSpacingSchedule 4.0 4.0 2.0 1.0)

(HowToCombineTransforms "Compose")

(NumberOfHistogramBins 32)

(ErodeMask "true")

(NumberOfResolutions 4)

(ImagePyramidSchedule 8 8 8 4 4 4 2 2 2 1 1 1)

(MaximumNumberOfIterations 2000)

(NumberOfSpatialSamples 2048)

(NewSamplesEveryIteration "true")

(ImageSampler "Random")

(BSplineInterpolationOrder 3)

(FinalBSplineInterpolationOrder 3)

(DefaultPixelValue 0)

(WriteResultImage "true")

(ResultImagePixelType "float")

(ResultImageFormat "nii")

(WriteIterationInfo "false")
