# Supplementary material for: Comparison of imaging-based bone marrow dosimetry methodologies and their dose–effect relationships in [177Lu]Lu-PSMA-617 RLT including a novel method with active marrow localization
Source: EJNMMI Phys. 2025 Dec 4;13:1. doi: 10.1186/s40658-025-00816-6 (PMC12779781; doi:10.1186/s40658-025-00816-6)
Supplement: Supplementary file 4 — Additional file4 (PDF 61 KB) [file 40658_2025_816_MOESM4_ESM.pdf]

ONLINE RESOURCE 4

In a voxel that does not contain tumor, the BVF and CF are computed as below:

$$BVF = \frac{\rho_{vox} - \rho_{YM} - \frac{m_{RM}}{v_{vox}}\left(1 - \frac{\rho_{YM}}{\rho_{RM}}\right)}{\rho_{bone} - \rho_{YM}}$$

$$CF = \frac{v_{RM}}{v_{RM} + v_{YM}} = \frac{\frac{m_{RM}}{\rho_{RM}}}{\frac{m_{RM}}{\rho_{RM}} + \frac{m_{YM}}{\rho_{YM}}} = \frac{\frac{m_{RM}}{\rho_{RM}}}{\frac{m_{RM}}{\rho_{RM}} + \frac{v_{vox}(\rho_{vox} - BVF * \rho_{bone}) - m_{RM}}{\rho_{YM}}}$$

$$= \frac{1}{1 - \frac{\rho_{RM}}{\rho_{YM}}\left(1 - \frac{v_{vox}(\rho_{vox} - BVF * \rho_{bone})}{m_{RM}}\right)}$$

With ρ, v, and m referring to the density, volume, and mass, respectively, of the whole voxel (vox), red marrow (RM), yellow marrow (YM), and bone ($bone$) constituting each voxel in the spongiosa. Note that ρ_{vox} is determined from the CT-derived density map; ρ_{RM}, ρ_{YM}, and ρ_{bone} are taken from ICRP 110; and m_{RM} is derived from [^{99m}Tc]Tc-sulfur colloid SPECT imaging (Equation 9).

In the four-compartment model that contains tumor, bone, yellow marrow, and red marrow we are assuming the volume of RM + YM is equal to the volume of tumor. As such we can find that the volume of tumor, v_t, is:

$$v_t = \frac{v_{vox}(\rho_{bone} - \rho_{vox}) + m_{RM}\left(1 - \frac{\rho_{YM}}{\rho_{RM}}\right)}{2\rho_{bone} - \rho_{YM} - \rho_t}$$

And given that the volume of YM is assumed to be $v_{YM} = v_t - v_{RM} = v_t - \frac{m_{RM}}{\rho_{RM}}$, we can compute the volume of bone in the voxel:

$$v_{bone} = \frac{\rho_{vox}v_{vox} - m_{RM} - \rho_t v_t - \rho_{YM}v_{YM}}{\rho_{bone}}$$

Thus, BVF and CF computed based on their definitions:

$$BVF = \frac{v_{bone}}{v_{vox}}$$

$$CF = \frac{v_{RM}}{v_{RM} + v_{YM}} = \frac{\frac{m_{RM}}{\rho_{RM}}}{v_t}$$

The voxel density, ρ_{vox} and red marrow mass, m_{RM}, are determined quantitatively from CT and $[^{99m}Tc]$Tc-sulfur colloid SPECT, respectively, but occasionally using the two values together in these equations resulted in non-physical determinations of the various spongiosa components (i.e. negative trabecular bone volume). This was mostly a problem in the case of the four-compartment model, where we assume the volume of tumor equals the volume of marrow, but this scenario is also possible in the three-compartment case. Instances where too much marrow was assigned to a voxel based on that voxel's CT-derived physical density were identified in pre-processing and excess marrow mass was moved to voxels that had available space, with the goal of preserving the total marrow mass of each delineated region as determined on $[^{99m}Tc]$Tc-sulfur colloid SPECT.
